# The potential and shortcomings of mitochondrial DNA analysis for cheetah conservation management

**DOI:** 10.1007/s10592-022-01483-1

**Published:** 2022-12-10

**Authors:** René Meißner, Sven Winter, Uta Westerhüs, Alexander Sliwa, Carola Greve, Lena Godsall Bottriell, Paul Bottriell, Carlos Rodríguez Fernandes, Paul Vercammen, Luke T. B. Hunter, Alexei V. Abramov, Leili Khalatbari, Petr Horin, Pamela A. Burger, Stefan Prost

**Affiliations:** 1 Research Institute of Wildlife Ecology, University of Veterinary Medicine, Savoyenstraße 1, 1160 Vienna, Austria; 2 Institute for Ecology, Evolution and Diversity, Goethe University, Max-von-Laue-Straße 13, 60438 Frankfurt am Main, Germany; 3 Opel-Zoo, von Opel Hessische Zoostiftung, Königsteinerstrasse 35, 61476 Kronberg im Taunus, Germany; 4 Kölner Zoo AG, Riehler Straße 173, 50735 Cologne, Germany; 5 LOEWE Centre for Translational Biodiversity Genomics (LOEWE-TBG), Senckenberganlage 25, 60325 Frankfurt am Main, Germany; 6 Rex Foundation, White Rock House, 2 Whipsnade Road, LU6 2NB Dunstable, UK; 7 CE3C - Centre for Ecology, Evolution and Environmental Changes & CHANGE - Global Change and Sustainability Institute, Departamento de Biologia Animal, Faculdade de Ciências, Universidade de Lisboa, 1749-016 Lisboa, Portugal; 8 Faculdade de Psicologia, Universidade de Lisboa, Alameda da Universidade, 1649-013 Lisboa, Portugal; 9 Breeding Centre for Endangered Arabian Wildlife, Sharjah, United Arab Emirates; 10 Wildlife Conservation Society, New York, NY USA; 11 School of Life Sciences, University of KwaZulu-Natal, Durban, South Africa; 12 Zoological Institute, Russian Academy of Sciences, Saint Petersburg, Russia; 13 Centro de Investigação Em Biodiversidade e Recursos Genéticos, CIBIO, InBIO Laboratório Associado, Campus de Vairão, Universidade Do Porto, Vairão, Portugal; 14 BIOPOLIS Program in Genomics, Biodiversity and Land Planning, CIBIO, Campus de Vairão, 4485‑661, Vairão, Portugal; 15 Mohitban Society, No. 91, Moghaddas Ardebili str., Tehran, Iran; 16 Department of Animal Genetics, University of Veterinary Sciences, Brno, Czech Republic; 17 Central European Institute of Technology, University of Veterinary Sciences Brno (CEITEC Vetuni), Brno, Czech Republic; 18 Konrad Lorenz Institute of Ethology, Savoyenstraße 1, 1160 Vienna, Austria; 19 Department of Behavioral and Cognitive Biology, University of Vienna, Djerassiplatz 1, 1030 Vienna, Austria; 20 Natural History Museum, Central Research Laboratories, Burgring 7, 1010 Vienna, Austria; 21 South African National Biodiversity Institute, National Zoological Garden, 232 Boom Street, 0002 Pretoria, South Africa

**Keywords:** Conservation genetics, Wildlife forensics, Phylogeography, Haplotype assignment, Subspecies assignment, Population genetics

## Abstract

There are only about 7,100 adolescent and adult cheetahs (*Acinonyx jubatus*) remaining in the wild. With the majority occurring outside protected areas, their numbers are rapidly declining. Evidence-based conservation measures are essential for the survival of this species. Genetic data is routinely used to inform conservation strategies, e.g., by establishing conservation units (CU). A commonly used marker in conservation genetics is mitochondrial DNA (mtDNA). Here, we investigated the cheetah’s phylogeography using a large-scale mtDNA data set to refine subspecies distributions and better assign individuals to CUs. Our dataset mostly consisted of historic samples to cover the cheetah’s whole range as the species has been extinct in most of its former distribution. While our genetic data largely agree with geography-based subspecies assignments, several geographic regions show conflicting mtDNA signals. Our analyses support previous findings that evolutionary forces such as incomplete lineage sorting or mitochondrial capture likely confound the mitochondrial phylogeography of this species, especially in East and, to some extent, in Northeast Africa. We caution that subspecies assignments solely based on mtDNA should be treated carefully and argue for an additional standardized nuclear single nucleotide polymorphism (SNP) marker set for subspecies identification and monitoring. However, the detection of the *A. j. soemmeringii* specific haplogroup by a newly designed Amplification-Refractory Mutation System (ARMS) can already provide support for conservation measures.

**Supplementary Information:**

The online version contains supplementary material available at 10.1007/s10592-022-01483-1.

## Introduction

The cheetah (*Acinonyx jubatus*, SCHREBER 1775) is one of the most remarkable yet unusual members of the family Felidae. Not only does its mostly diurnal lifestyle deviate from most other wild felids, but it is also the fastest carnivore and reaches the highest speed of any land animal of up to 105 km/h (Sharp 1997). Due to its uniqueness, the species inhabits a distinct niche within African and Asian ecosystems, with distinctive subspecies occurring in different habitats (Mills et al. 2004). Adapted to high-speed running, their main source of prey consists of small to medium-sized antelopes that are captured by short-distance sprints (Mills et al. 2004). Hunting smaller prey than lions, leopards, and hyenas, cheetahs avoid interactions with the larger carnivores, one of their major natural threats (Durant 2000; Hunter et al. 2007). Historically, cheetahs inhabited most parts of Africa (excluding tropical rainforests), the Arabian Peninsula, and large parts of Southwest Asia (Durant et al. 2017). Presently, their distribution consists only of a small fraction of the species’ former range, and most populations are isolated from each other (Fig. 2a; Durant et al. 2017). The International Union for Conservation of Nature (IUCN) Cat Specialist Group currently recognizes four cheetah subspecies: the Southern African cheetah (*Acinonyx jubatus jubatus*); the Asiatic cheetah (*A. j. venaticus*); the Northeast African cheetah (*A. j. soemmeringii*) and the Northwest African cheetah (*A. j. hecki*) (Kitchener et al. 2017). Traditionally the cheetah was divided into five subspecies (Krausman and Morales 2005). However, the East African cheetah (*A. j. raineyi*) has been recently subsumed into the Southern African cheetah (*A. j. jubatus*) due to a non-monophyletic clustering on the mtDNA level (Charruau et al. 2011; Kitchener et al. 2017), a change since challenged (Prost et al. 2022). Here, we follow the classical five subspecies concept from Krausman and Morales (2005) while acknowledging that *A. j. raineyi* is currently not accepted as a fifth subspecies by the IUCN. The species as a whole is listed as “vulnerable” by the IUCN, with approximately 7,100 adult and adolescent individuals left in the wild (Durant et al. 2017), showing a drastic reduction in population size compared to the estimated 15,000 individuals 45 years ago (Myers and Resources 1975). The subspecies *A. j. venaticus* and *A. j. hecki* are already listed as “critically endangered” (Durant et al. 2017), and, with fewer than 50 individuals, *A. j. venaticus* is likely to face extinction in the near future (Durant et al. 2017; Farhadinia et al. 2016, 2017; Khalatbari and Yusefi et al. 2018). Thus, while the species as a whole is likely to survive, current conservation efforts, e.g., reintroduction of cheetahs into former range countries, should take subspecies assignments into account as the loss of certain subspecies might irreversibly alter the corresponding ecosystems (Bertola et al. 2022; Liu et al. 2018).

Multi-factorial causes affect cheetah population declines and can be divided into manmade causes such as habitat loss, illegal wildlife trade (Klaassen and Broekhuis 2018; Marker and Dickman 2004), climate change (Khalatbari and Jowkar et al. 2018), and non-manmade causes, mainly related to inbreeding (Merola 1994; O’Brien et al. 1985, 1986). In recent years, illegal trafficking especially for the exotic pet trade has become a major threat to cheetahs (Tricorache et al. 2018). This issue is further intensified by the political instability in many of the cheetah’s range countries (Brito et al. 2018; Jacobs and Schloeder 2001). As the species is listed in Appendix I of the Convention on International Trade in Endangered Species of Wild Fauna and Flora (CITES), the legal trade of captive-bred cheetahs is strongly regulated, and any trade with wild-caught individuals is banned with few exceptions (https://cites.org/eng/app/appendices.php). In most countries within the species’ range, the cheetah is protected by law and different conservation plans act to ensure legislation (Balint et al. 1997; Nowell and Jackson 1996). Cheetah trade is of high economic value, which creates a black market for illegal trading and organized poaching of live cheetah cubs (Magliolo et al. 2021). The main customers of exotic pets are primarily found within the Arabian Peninsula where a pet cheetah counts as a status symbol. In many Arabic cultures, hunting with captive cheetahs has a long tradition, and the pet trade might be a relic of those habits (Spee et al. 2019). Poaching of live cheetahs occurs in different intensities throughout their range and therefore affects the subspecies differently. With *A. j. raineyi* and *A. j. soemmeringii* close to one of the major illegal trading hubs, the Horn of Africa, poaching is an increasing threat, especially in countries such as Ethiopia, Somalia, and Kenya (Tricorache et al. 2021). To monitor illegal trade and to identify poaching hotspots, identifying cheetah subspecies and reliably detecting each individual’s geographical origin is crucial. In the case of East Africa, delineating the subspecies could reveal whether an individual was poached in Ethiopia, South Sudan, Northern Somalia, or in Kenya, Tanzania, Uganda, or Southern Somalia, as these are the respective distributions of the two subspecies, *A. j. soemmeringii* and *A. j. raineyi*.

It is challenging to distinguish cheetah subspecies morphologically, and they are often assigned to subspecies-level solely based on their geographic origin. However, cheetahs have been recorded to move > 1000 km (Durant et al. 1988; Farhadinia et al. 2016). Genetics has shown promise to effectively identify cheetah subspecies (Charruau et al. 2011; Schmidt-Küntzel et al. 2018; Prost et al. 2022). In general, it has been shown that mitochondrial DNA (mtDNA) barcodes can reliably discriminate between species (Tavares and Baker 2008) and even subspecies (Soares et al. 2019; Gaber et al. 2020). However, several conflicting signals regarding mitochondrial DNA-based subspecies identification in cheetahs have been identified. For example, *A. j. raineyi* and *A. j. jubatus* have recently been merged into a single subspecies, namely *A. j. jubatus*, based on mtDNA data (Kitchener et al. 2017). Yet, this finding does not fit recent evidence obtained from genome-wide SNP data (Prost et al. 2022), raising questions about the validity of mtDNA markers for subspecies assignment in cheetahs. In their study, Prost et al. (2022) suggest incomplete lineage sorting (ILS) or mitochondrial capture (MC) as possible reasons for the presence of two mitochondrial haplogroups in East African cheetahs, one of which is shared with the Southern African subspecies, *A. j. jubatus*. ILS is the retention of two or more alleles originating from an ancestral population in one or two populations after divergence/speciation (Hahn 2019). MC can be caused by mitochondrial introgression from one population into another, if the introgressed mitochondrial genome fixates in the receiving population (Allendorf et al. 2022). Furthermore, mitochondrial and nuclear DNA discordance is a well-known phenomenon in many taxa (Toews and Brelsford 2012).

To obtain better insights into the mitochondrial phylogeography of cheetahs and to investigate potential polymorphisms for subspecies identification, we used 15 mitochondrial SNPs and a 3-bp deletion in the NADH dehydrogenase subunit 5 (ND5; Charruau et al. 2011 and Prost et al. 2022), amplified in five short mtDNA mini-barcodes (cheetah subspecies specific amplicons; CSAs), to infer the presence of mitochondrial haplotypes/haplogroups throughout the cheetah’s current and former range. The CSAs, not exceeding 200 bp in length, were chosen to enable amplification even in highly degraded samples e.g., museum or confiscated material. Three of these amplicons have previously been used in Prost et al. (2022). Using an extended CSA and sample set we show that differences exist across mtDNA haplogroups, but that especially in East Africa, subspecies assignment using only mtDNA is confounded by the presence of two mitochondrial haplogroups. Furthermore, as the Horn of Africa is a poaching hotspot, we designed an easy-to-use PCR approach (Amplification-Refractory Mutation System; ARMS) for the identification of the *A. j. soemmeringii* haplogroup, which does not require sequencing of the amplicons.

## Methods

### Sample collection

Within this study, we used 230 historic and 33 contemporary cheetah samples consisting mostly of skin and bone tissue (see Supplementary Table S1). Our sample set covers most of the species’ former and current range throughout Africa and southwest Asia. We included all five traditionally recognized subspecies (assigned to subspecies based on geographical origin), more precisely, 75 *A. j. jubatus*, 39 *A. j. raineyi*, 51 *A. j. soemmeringii*, 51 *A. j. venaticus*, 31 *A. j. hecki* and 16 individuals of unknown origin. We also included samples previously used in Prost et al. (2022). A complete list of all 263 individual samples with detailed information, including origin, age, and conducted analysis, can be found in Supplementary Table S1. Samples collected after 1975 were imported under the following CITES numbers: AT 16-E-0753, 16SG006329CR, 15JP001990/TE, 11US761881/9, AT 15-E-1769, D79/DFF or transferred between different CITES-registered institutions (Supplementary Table S2).

### DNA extraction

To extract genomic DNA, we used the QIAamp® Fast DNA Tissue Kit (QIAGEN N. V., Hilden, North Rhine-Westphalia, Germany) or a DNA salting out method (De Volo et al. 2008). Prior to extended lysis overnight, we rehydrated the samples in nuclease-free water for 24 h in an attempt to remove potential secondary substances used in sample preservation.

#### Cheetah subspecies-specific amplicons (CSAs)

The Cheetah Subspecies-specific Amplicons (CSAs) are short mtDNA barcodes that contain specific SNPs, which allow discrimination between the five identified cheetah mitochondrial haplogroups. We designed six CSAs (Table 1) that include a total of 17 relevant SNPs and one 3-bp deletion (two were later excluded with CSA-6, see below), three of which were previously applied in Prost et al. (2022). A combination of all six amplicons enables an unambiguous mitochondrial haplogroup assignment, and, depending on the haplogroup, a single SNP can already be diagnostic.

**Table 1:** Primer list of the Cheetah Subspecies-specific Amplicons (CSAs), including the location on the mitochondrial genome [mt location], primer orientation, primer sequence, annealing temperature [TM[°C]], and length of the amplicon, including the universal Illumina tails (p5 + p7 = 66 bp) [length [bp]]

Amplicon ID.	mtDNA region	orientation	sequence 5’-3’	T_M_ [°C]	length [bp]
AMP1	ND5	forward	TGCAACTCCAAATAAAAGTAATAAA	55	130 + 66
		reverse	TTTACATAGTGGGGGTATAAGTTG		
AMP2	ND5	forward	CCCCCGTAGCACTATTTGTT	55	80 + 66
		reverse	TCGGTTAATGTATGGATCTGAGTG		
AMP3	ND5	forward	TCCAACTATTTATTGGCTGAGAA	55	76 + 66
		reverse	TCTGTTCGGCCATATCATCA		
AMP4	CR	forward	CACTTCCAACAAAACAAACCAA	55	90 + 66
		reverse	GAGACGGGGTGGTTGATAGA		
AMP5	CR	forward	CGGGACAATTCTCTATGGAC	55	101 + 66
		reverse	GGAGCGAGAAGAGGTACACG		
AMP6	CR	forward	CCTGGCATCTGGTTCTTACC	55	66 + 66
		reverse	TTTGAGAAAGTTGAAGGATTGGA		

We based the SNP set on an alignment of 929 bp of ND5 and the Control Region (CR) from 53 cheetahs, including 18 *A. j. jubatus*, five *A. j. raineyi*, 17 *A. j. soemmeringii*, ten *A. j. venaticus*, three *A. j. hecki*, and five individuals of unknown subspecies (Prost et al. 2022; Dryad: https://doi.org/10.5061/dryad.tx95 × 6b13 ). To examine the sequences manually and select the SNPs, we used the freely available alignment software BioEdit v7.0.5 (http://www.mbio.ncsu.edu/BioEdit/bioedit.html).

A region was considered as an appropriate CSA when fulfilling the following requirements: (1) regions include SNPs that are absent in at least one subspecies or, preferentially, unique to a single subspecies; (2) regions show no variation within the chosen subspecies subset, and (3) produce amplicons at least 75 bp in length, but not longer than 200 bp; (4) primers needed to have the same or similar annealing temperatures and no overlap in sequences to enable pooled amplification of all CSA primers in a single reaction. We added universal tails for Illumina sequencing (p5 forward: 5’-ACACTCTTTCCCTACACGACGCTCTTCCGATCT-3’ and p7 reverse: 5’-GTGACTGGAGTTCAGACGTGTGCTCTTCCGATCT-3’) to the primers 5’-ends to enable next generation sequencing on an Illumina platform of the amplicons; (5) forward and reverse primers are universal to the given 53 mitochondrial sequences and ideally 20 bp in length. To enable primer universality, we allowed base pair ambiguities except for the first 3 bp at the 3’-end, as this would significantly reduce primer efficiency (Newton et al. 19891989). For PCR 1, the primer ligation multiplex PCR, we used 6.25 µl QIAGEN Multiplex PCR Master Mix (QIAGEN GmbH, Hilden, Germany), 0.5 µl of each of the 12 primers with a concentration of 10 µM (see Table 1) and 0.3 µl of non-acetylated bovine serum albumin (50 mg/mL); Template DNA varied between < 1 ng/µl and 10 ng/µl depending on sample quality; ddH_2_O was added to a total reaction volume of 15 µl. PCR 1 followed standard settings with an annealing temperature of 55 °C and 38 cycles. For PCR 2, the Illumina adapter ligation PCR, we used a total reaction volume of 15 µl, with 10.25 µl x10 PCR-Buffer b (Solis BioDyne, Tartu, Estonia), 1.25 µl MgCl (2 mM), 1.25 µl dNTPs (2 mM), 0.5 µl non-acetylated bovine serum albumin (50 mg/mL), 0.25 µl FIREpol DNA Polymerase (5 U/µl, Solis BioDyne, Tartu, Estonia) and 0.25 µl of the p5 and p7 illumina adapters. For each sample, a unique adapter combination was used. All used adapters with indices are listed in Supplementary Table S3). We used 1 µL of the PCR product of PCR 1 as template for PCR 2. PCR 2 followed standard settings with an annealing temperature of 55 °C and 5 cycles.﻿ .

#### Illumina sequencing

We conducted the sequencing on an iSeq 100 system (Illumina, Inc., San Diego, California, USA). The library preparation and the Illumina adapter ligation PCR followed Lange et al. (2014). Amplification and adapter ligation were carried out in a single tube for each sample (Supplementary Table S1). We pooled all samples before purification and eluated the pooled library in 1 M Tris. The iSeq 100 system was loaded with 20 µl of 100 pM diluted library solution. The sequencing run used the ‘IDT-ILMN TruSeq DNA-RNA UD Indexes 96 Indexes’-program for barcoding approaches.

### Bioinformatic analyses

We used Illumina’s bcl2fastq version 2–20 (https://emea.support.illumina.com/sequencing/sequencingsoftware/bcl2fastq-conversion-software.html) software for basecalling of the raw read data and adapter trimming with default settings. We utilized the Unix grep command for sample de-multiplexing based on the specific sample index combinations contained in the read header. We mapped the reads to the cheetah mitochondrial reference genome (NC_005212.1) using BWA mem (Li and Durbin 2010) with default parameters. The mapped reads were sorted with SAMtools sort (Li et al. 2009), and the consensus was called with angsd version 0.935-33-g79d9455 (Korneliussen et al. 2014) using the flags ‘–doFasta 2’ and ‘–doCounts 1’.

We reconstructed medium-joining networks based on (i) the complete CSA sequences and (ii) the reduced CSA 15 SNPs and 3 bp deletion data using the freeware tool PopART v. 1.7 (Bandelt et al. 1999). Next, we performed a Discriminant Analysis of Principal Components (DAPC) (Jombart and Collins 2017) with the package ‘adegenet’ (Jombart 2008) in ‘R’ version 2022.2.0.443 (RStudio Team 2022) using the 15 SNPs and 3-bp deletion of the CSAs from 153 individuals to define haplogroups. We predefined 5 clusters based on (i) the geographical origin of the individuals and (ii) the identified mtDNA haplogroups. To convert our sequencing data into a ‘genind’ class object (required as input for the DAPC), we used the ‘read.FASTA’ and ‘as.matrix’ functions of the ‘ape’ package (Paradis and Schliep 2019) followed by the ‘adegenet’ function ‘DNAbin2genind’. We retained four principal components and three discriminant functions. We plotted the DAPC results using the ‘scatter’ function of the ‘ggplot2’ package (Wickham et al. 2016) and the ‘compoplot’ function (‘adegent’). All ‘R’ packages used for the cluster analysis can be freely downloaded at: http://cran.r-project.org/web/packages/.

#### Amplification refractory mutation system (ARMS)

Based on the results of our sequencing approach, we designed an Amplification Refractory Mutation System (ARMS). Unfortunately, preliminary testing showed that the original approach based on Newton et al. (1989) was not stringent enough to use single nucleotide differences to distinguish between the different cheetah haplogroups. Even when we included artificial mismatches at the primers 3’-end as suggested by the authors, we could not enable specific primer refraction. Therefore, we instead created one diagnostic ARMS for the *A. j. soemmeringii* haplogroup based on a 3-bp deletion in ND5. Further, we altered the system of Newton et al. (1989) to utilize mtDNA markers instead of nuclear DNA by including an additional third primer (Table 2). Both outer primers amplified all cheetah subspecies, and only the single inner primer was specific to the *A. j. soemmeringii* haplogroup. Hence, the ARMS-PCR was designed to produce (1) two amplicons of different lengths for the *A. j. soemmeringii* haplogroup and (2) one amplicon for all other cheetah haplogroups. Gel-electrophoresis on a 4% agarose gel allowed the clear differentiation of the *A. j. soemmeringii* haplogroup based on the number and length of visible bands without additional sequencing.

**Table 2:** ARMS specifications including type of PCR-refraction, location of the refraction side on the mitochondrial genome, primer orientation, primer sequence (>< = 3-bp deletion), annealing temperature [TM[°C]] and length of the amplicon [length [bp]]

type		mtDNA location	primer	sequence 5’-3’	TM [°C]	bp
mismatch		mt12665-	outer forwards	GGCTTTTTCAACTTTTATAGGAT	55	189
		mt12667	outer reverse	TTTACATAATGGGGGTATAAGTTG	55	
			inner reverse	CAGTTAGTGTAAATGAGGTA > < AAG	55	110

***Supplementary Material***

Supplementary Fig. 1 visualizes the SNP data of the whole CSA in a medium-joining network (excluding CSA6). Supplementary Table 1 includes the complete sample list of all 263 individuals used in this study with detailed information. Supplementary Table 2 lists the illumina adapters for the CSA sequencing approach. Supplementary Table 3 shows the presence/absence of the three possible bands of tested samples occurring on agarose gel of the *A. j. soemmeringii* haplotype specific ARMS. Supplementary Table 4 lists the CITES-registered institutions within this study and their registration numbers

## Results

Our final dataset included 153 individuals out of the 263 tested historic samples. The first filtering step discarded samples with highly degraded or too little DNA yield to be amplified by the adapter PCR. From those 220 samples passing this pre-sequencing quality check, we obtained sequence reads for 180 individuals. Additional 27 samples were removed from the dataset as they showed signs of contamination. Furthermore, we removed CSA-6 from the dataset, as this amplicon showed the highest amount of contamination while not adding much information to the haplotype assignments. All DNA extraction controls and PCR blanks were negative.

### Mitochondrial phylogeography of the cheetah subspecies

**Fig. 1:**
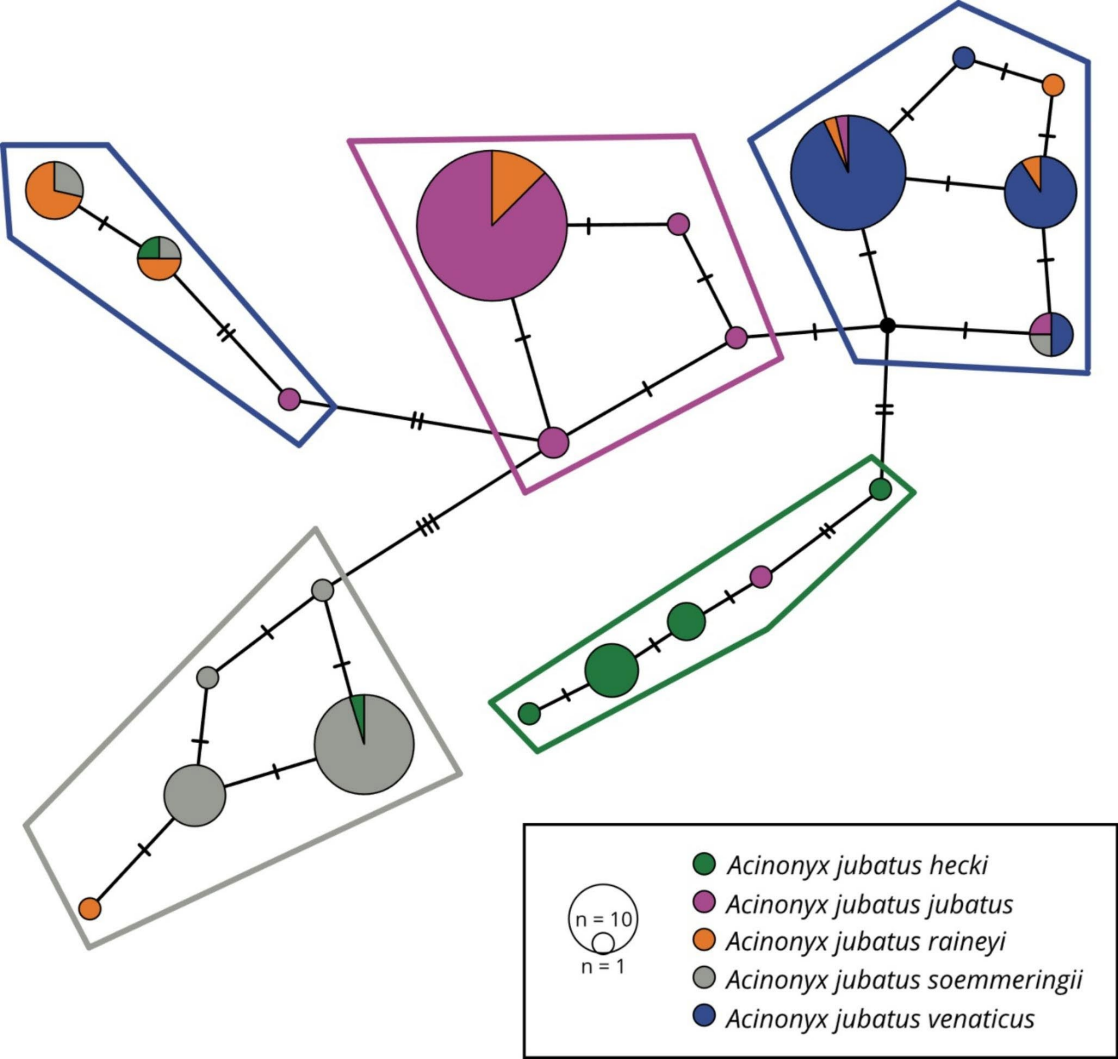
Median-joining network for 153 cheetah individuals based on the discriminatory 3-bp deletion and 15 SNPs of the Cheetah Subspecies-specific Amplicons (CSAs). Pie-chart diameters represent sample sizes supporting a specific haplotype. Colors represent subspecies assignment based on sample origin. We highlighted subspecies haplogroups, and mutations between haplotypes are indicated by dashes

The phylogeographic analyses using medium-joining networks showed five distinct clusters corresponding largely to the five classically recognized subspecies, *A. j. hecki*, *A. j. jubatus*, *A. j. raineyi*, *A. j. soemmeringii*, *A. j. venaticus* (Fig. 1; Supplementary Figure S1). In general, few deviations between geographic origin-based and CSA-based subspecies assignment were found. Individuals assigned to *A. j. raineyi* based on their geographic origin were dichotomous and nested either in the *A. j. jubatus* haplogroup or formed their own *A. j. raineyi* haplogroup. This haplogroup also included several individuals from southern Somalia and southern Ethiopia, previously described as the border between the two subspecies *A. j. raineyi* and *A. j. soemmeringii* (Charruau et al. 2011; Prost et al. 2022). As shown before (Prost et al. 2022), samples AJ164 from Zimbabwe and AJ028 from Tanzania showed haplotypes close to the *A. j. venaticus* haplogroup. In addition, we found a second Tanzanian sample (AJ250) exhibiting an *A. j. soemmeringii* haplotype.

Compared to the medium-joining networks based on the complete CSA sequences (Supplementary Figure S1), we did not lose much haplotype information, which supports the discriminatory power of our reduced 3-bp deletion and 15 SNP approach.

**Fig. 2:**
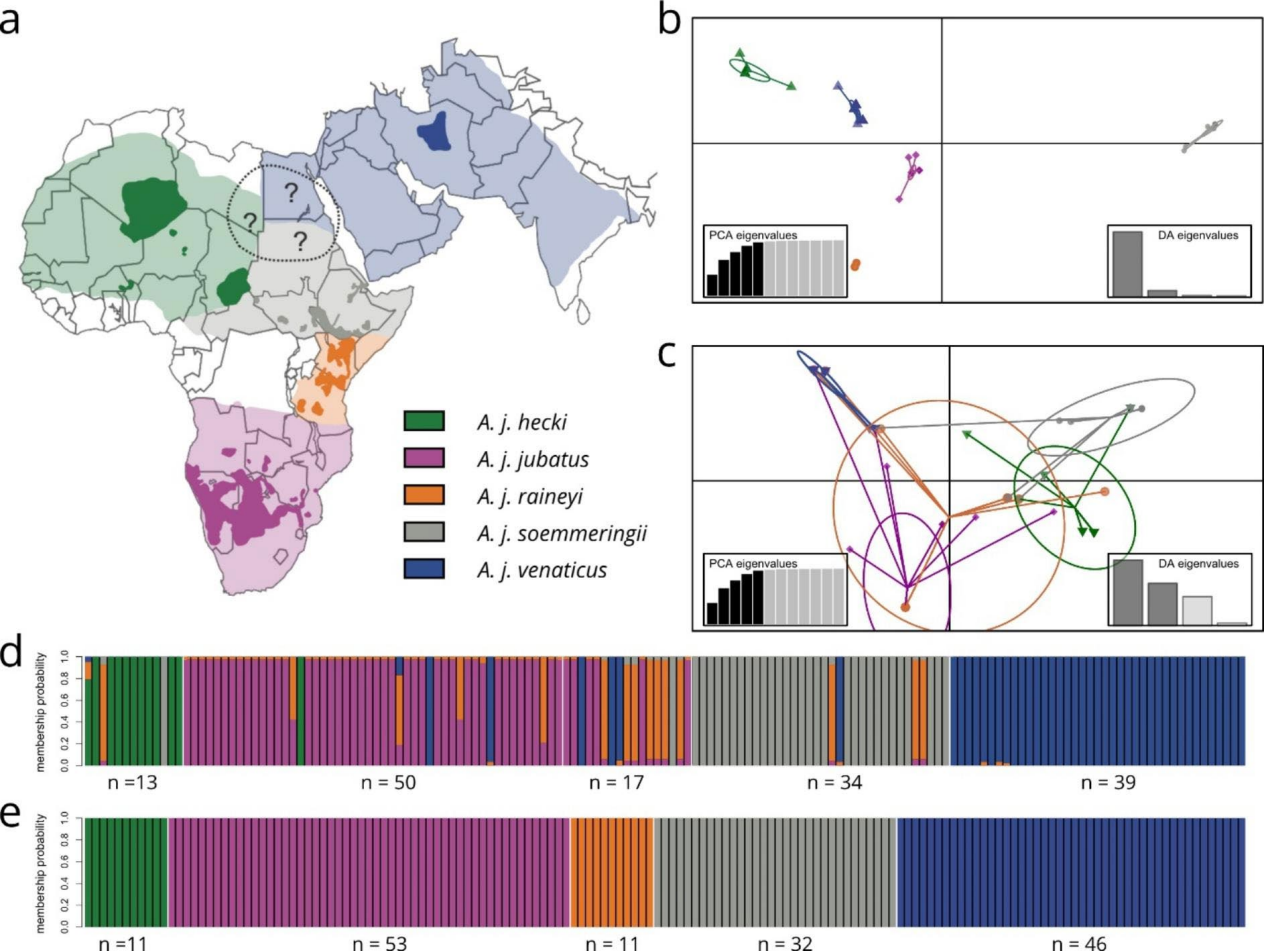
Phylogeographic distribution and population structure based on mitochondrial DNA. a) Map showing expected historic (light colors) (Marker 2019) and current distribution (dark colors) (Durant et al. 2017) of the five cheetah subspecies (Acinonyx jubatus ssp.). b and c) Scatterplots showing the coordinates for the individuals (retained principal components of the DAPC) plotted on the principal axis of the DAPC (discriminant functions) using the 15 SNPs and 3-bp deletion of the CSAs from 153 individuals suggesting 5 clusters based on (b) haplogroup-based subspecies assignment or (c) geographical origin-based subspecies assignment. The cumulated variance of the PCA and the discriminative analysis (DA) eigenvalues, are shown as in-figure barplots respectively. d and e) Results of the DAPC membership probability analysis based on (d) geographical origin-based subspecies assignment and (e) haplogroup-based subspecies assignment. Each vertical line represents a single individual. Colored segments indicate the individual’s estimated proportion of membership to the haplogroups

Next, we investigated haplogroup structure within our data set using DAPC. This analysis confirmed the distinction into five haplogroups (Fig. 2b) but showed discrepancies between haplogroup-based and geographic origin-based subspecies identification (Fig. 2c). Similarly, we identified assignment difficulties when we calculated membership probabilities for the different individuals to the five haplogroups (Fig. 2d) based on their geographic origin. However, we had good discrimination power for the five haplogroups when individuals were assigned to the haplogroups based solely on their genetic profile (Fig. 2e*).*

**Fig. 3:**
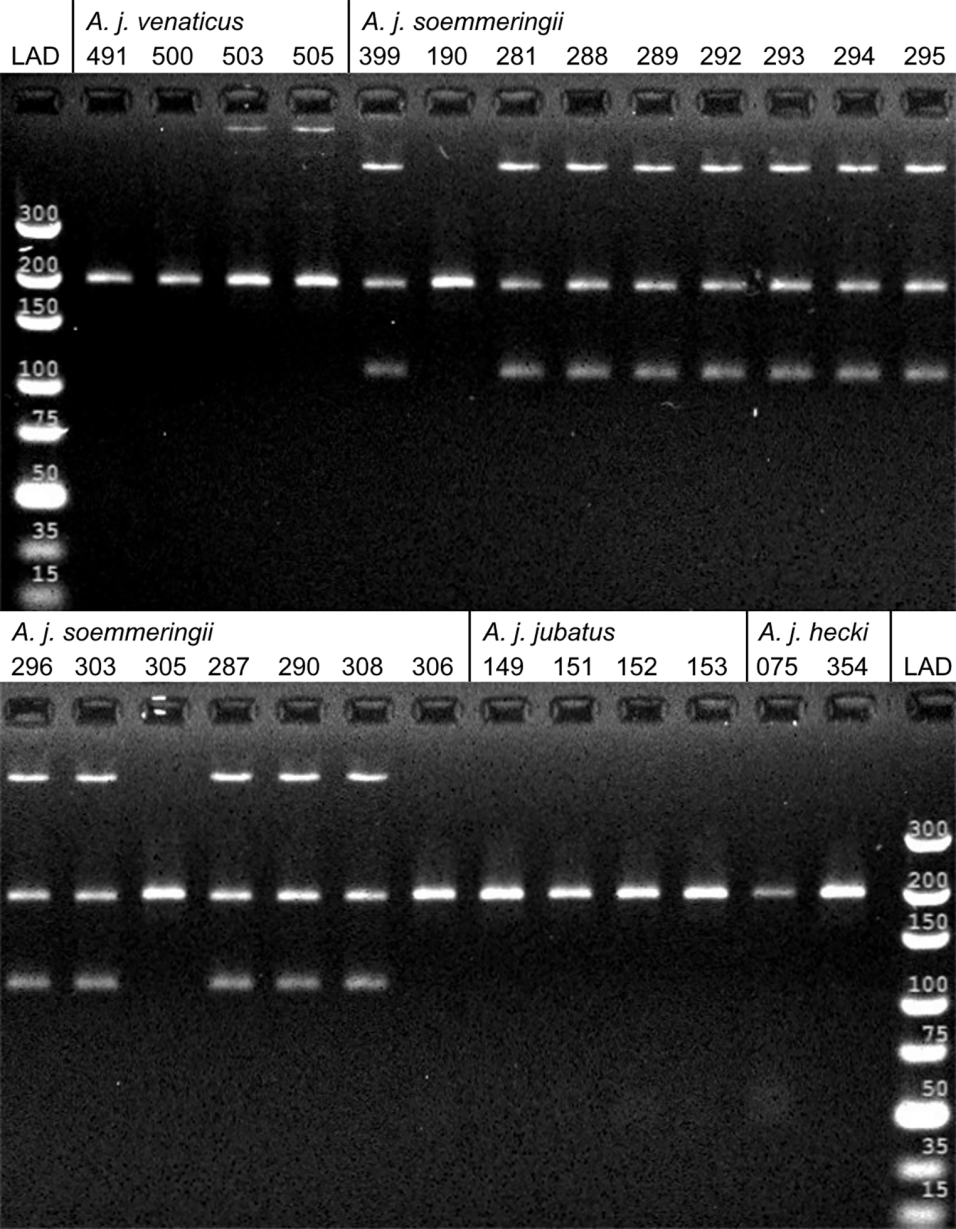
Electrophoresis results of the *A. j. soemmeringii* ARMS PCR in 26 cheetahs. 189 bp control amplicon and 110 bp *A. j. soemmeringii*-specific amplicon. Cheetah subspecies were assignment based on geographic origin. LAD = GeneRuler Ultra Low Range DNA Ladder. Individual 190 originates from southern Ethiopia, the border of *A. j. soemmeringii* and *A. j. raineyi*. It was tentatively assigned to *A. j. soemmeringii*, but probably belongs to *A. j. raineyi*

A simple genetic approach to identify individuals belong to the *A. j. soemmeringii* haplogroup based on mtDNA.

Using a three-primer approach, we created a functional ARMS-PCR system that can reliably identify the *A. j. soemmeringii* haplogroup-specific 3-bp deletion. Interestingly, our system resulted in three visible bands for samples with an *A. j. soemmeringii* haplotype instead of the expected two bands. In-silico, we investigated all publicly available cheetah nuclear and mitochondrial genomes for nuclear mitochondrial DNA segments (NUMTs) or other structures that might cause the observed third band. We did not discover any potential PCR products matching the length of the observed additional band. However, no *A. j. soemmeringii* genome is available at the moment, so we could not test whether this might be due to some subspecies-specific NUMTs. All individuals carrying other mitochondrial haplotypes only showed one band after amplification as expected, and the results of the ARMS-PCR matched our Illumina sequencing data. Samples AJ305 and AJ306 displayed an *A. j. raineyi* mitochondrial haplotype in the Illumina sequencing data, but clearly belonged to *A. j. soemmeringii* based on the genomic data (Prost et al. 2022). Individual AJ190 originated from Southern Ethiopia, the border of the *A. j. soemmeringii* and *A. j. raineyi* ranges. It was tentatively assigned to *A. j. soemmeringii*, but based on the ARMS approach it likely belongs to *A. j. raineyi*. However, we cannot be certain as it still could be assigned to *A. j. soemmeringii* based on nuclear data, similar to AJ305 and AJ306 (Prost et al. 2022). For a detailed list of all samples tested with the ARMS approach and the results, see Supplementary Table S4.

## Discussion

Mitochondrial phylogeography, and subspecies assignment based on mtDNA.

With our CSAs not exceeding 130 bp, contemporary and highly degraded historic samples could be amplified, with the oldest specimen in our sampling dating back to 1815. With only 15 relevant SNPs and one 3-bp deletion, we were able to detect haplotypes belonging to five distinct haplogroups. In general, these haplogroups corresponded to the five traditionally recognized cheetah subspecies, with *A. j. raineyi* harbouring two haplogroups (Charruau et al. 2011; Prost et al. 2022). However, our results support findings that solely relying on mtDNA for subspecies identification or conservation unit assignment might not be sufficient in all cases. It is thus crucial to distinguish between belonging to a mitochondrial haplogroup or subspecies. Our analyses on an extended data set, together with previous findings (Charruau et al. 2011; Prost et al. 2022), indicate a much more complex maternal history in parts of the species’ distribution range. Particularly, individuals in East Africa showed either *A. j. jubatus* or *A. j. raineyi* mitochondrial haplotypes with no apparent geographical structure. Similar to East Africa, the evolutionary history of cheetahs in Northeast Africa seems to be more complex than previously thought. Applying a subset of the CSA’s used in this study, Prost et al. (2022) found two individuals that belonged to the *A. j. soemmeringii* subspecies based on genome-wide data and geographic origin-based assignment that carry *A. j. raineyi* mitochondrial haplotypes. Similarly, with the extended CSA sampling, these two individuals (AJ305 and AJ306) showed an *A. j. raineyi* mitochondrial haplotype. Furthermore, the presence of some *A. j. soemmeringii* individuals carrying *A. j. raineyi* mitochondrial haplotypes hints toward ILS or MC as potential evolutionary forces behind these findings. In addition, we found three individuals carrying *A. j. raineyi* mitochondrial haplotypes that originated in the far south of Ethiopia and Somalia (AJ189, AJ190, AJ191). Unfortunately, we did not have genomic data for these individuals to understand whether they would assign to *A. j. raineyi* or *A. j. soemmeringii* based on their nuclear DNA. It will be important to clarify which regions are exclusively inhabited by one subspecies and in which areas both subspecies may co-occur. Given the possibility that *A. j. soemmeringii* individuals from Northeast Africa carry *A. j. raineyi* mitochondrial haplotypes and that the exact border between the two subspecies is unknown complicates the subspecies assignment of individuals carrying *A. j. raineyi* mitochondrial haplotypes. Within the *A. j. soemmeringii* haplogroup we only found two conflicting samples, AJ250 from northern Tanzania and AJ356 from southern Libya. AJ356 was assigned to *A. j. hecki* according to its regional origin as it is currently assumed that Libya falls within *A. j. hecki*’s former range (Charruau et al. 2011; Prost et al. 2022). Apart from ILS or MC, one possible explanation for the *A. j. soemmeringii* mitochondrial haplotype detected in the individual from southern Libya might be the cheetah’s ability to travel long distances. Cheetah home ranges are often very large with movements exceeding 1000 km (Durant et al. 1988; Farhadinia et al. 2016), therefore, similar inconsistencies between haplotype and geographical origin within our data set might be related to long distance migration. Furthermore, before their widespread local extinctions, the distribution ranges of *A. j. soemmeringii* and *A. j. hecki* might have been much closer than they are today. Similarly, the Tanzanian sample AJ250 showing an *A. j. soemmeringii* haplotype could also be explained by long-distance travel.

All geographical origin-based *A. j. jubatus* individuals show mitochondrial haplotypes of the *A. j. jubatus* haplogroup. However, *A. j. jubatus* haplotypes are not exclusive to this subspecies, but also occur in cheetahs from East Africa (*A. j. raineyi*), which seem to be different from *A. j. jubatus* on the nuclear DNA level (Prost et al. 2022) making subspecies assignment based solely on this haplotype impossible.

Finally, we detected two *A. j. venaticus* haplotypes in samples from Chad, which is thought to be within *A. j. hecki*’s distribution. The presence of *A. j. venaticus* mitochondrial haplotypes in North Africa (in Egypt) has already been indicated in Charruau et al. (2011). Given the close proximity of Egypt to *A. j. venaticus*’s West Asian distribution, it is possible that the distribution of this subspecies ranged into North Africa. However, to see whether it reached all the way to Chad requires further sampling and analysis.

### Illegal trade monitoring

Given the importance of the Horn of Africa in the illegal international trade of wild cheetahs, reliable subspecies identification could help to identify poaching hotspots. Cheetahs traded via Somalia and Yemen to the Gulf states are thought to originate either from Ethiopia or Kenya (Tricorache et al. 2018). East Africa is the contact zone between the two subspecies: *A. j. raineyi* and *A. j. soemmeringii* (Durant et al. 2017). While *A. j. soemmeringii* is thought to inhabit South Sudan, the northern and central parts of Ethiopia and Somalia, *A. j. raineyi* occurs in Kenya, Tanzania, Uganda, and the southern parts of Ethiopia and Somalia. Assigning confiscated cheetahs at the Horn of Africa, e.g., to the *A. j. soemmeringii* haplogroup using a simple and cost-effective ARMS approach could indicate whether confiscated individuals come from the north of the contact zone or the south. Our tests showed the ARMS approach’s reliable detection of the *A. j. soemmeringii* haplogroup-specific 3-bp deletion. Unfortunately, even when artificial mismatches were included, the ARMS approach was not stringent enough to detect single nucleotide differences in other subspecies. Thus we were not able to develop an ARMS approach for other subspecies. The developed ARMS allows laboratories to inexpensively screen large numbers of samples, as well as single specimens, for the presence of the *A. j. soemmeringii* haplogroup, avoiding more complicated and expensive approaches such as Sanger or high-throughput sequencing.

### Limitations with museum samples

In general, we cannot exclude potential identification problems related to our samples, as many originated from museum collections and several samples exceeded 150 years of storage history. For instance, AJ164 from Zimbabwe showed an *A. j. venaticus* haplotype instead of the expected *A. j. raineyi* or *A. j. jubatus* haplotypes. In addition to the previously discussed possibilities of ILS and MC in cheetah subspecies, mislabeling during the samples’ individual storage history could be a likely explanation. Another related issue with museum material is contamination, which can occur either during storage or sample handling. We avoided cross-contamination in the laboratory by working in a dedicated clean room, monitoring possible contamination using extraction and PCR blanks. Furthermore, we excluded samples with more than one mitochondrial haplotype after sequencing. As most of our samples contained DNA concentrations below 1 ng/µl, even the slightest contamination might be picked up during the PCR amplification. However, not a single extraction or PCR blank showed any sign of amplification. Thus, the detected contaminations likely originated from the samples themselves and included other cheetah subspecies and feline species such as the leopard, or completely unrelated carnivores like the red fox. The detection of these contaminants highlights the difficulties of working with historic material. Therefore, the future inclusion of more modern samples will be essential to validate the detected haplotype structure.

## Conclusion

Our study shows that mtDNA alone might not be a sufficient indicator for a cheetah’s geographic origin and subspecies assignment. Our analyses, along with previously published studies (Charruau et al. 2011; Prost et al. 2022) found inconsistencies between geographic origin and mtDNA-based subspecies assignments and the presence of more than one mitochondrial haplogroup in a single cheetah subspecies. Furthermore, cheetah show mitochondrial and nuclear DNA discordance, which has previously been recorded for several other taxa (reviewed in Toews and Brelsford 2012). This highlights the need to treat inferences and conservation decisions based solely on mtDNA with caution. However, we also outlined and discussed the use of mtDNA for targeted questions, such as identifying *A. j. soemmeringii* haplotypes to monitor the illegal cheetah trade in the Horn of Africa. Our analysis supports the great need for a combination of nuclear and mtDNA data to answer complex phylogeographic and phylogenetic questions (Toews and Brelsford 2012). Further studies on cheetahs must include both nuclear and mtDNA data from all cheetah subspecies to assign individuals to conservation units, inform subspecies-based conservation measures, and aid subspecies identification in a wildlife forensic context.

## Electronic supplementary material

Below is the link to the electronic supplementary material.

Supplementary Material 1

Supplementary Material 2

## Data Availability

All mtDNA sequence data used in this study are provided in Supplementary File S2 and on dryad (10.5061/dryad.b8gtht7h1).
