# Supplementary material for: The potential and shortcomings of mitochondrial DNA analysis for cheetah conservation management
Source: Conserv Genet. 2022 Dec 10;24(1):125–36. doi: 10.1007/s10592-022-01483-1 (PMC9859914; doi:10.1007/s10592-022-01483-1)
Supplement: Supplementary file 1 — Supplementary Material 1 [file 10592_2022_1483_MOESM1_ESM.pdf]

Supplementary Material

"The potential and shortcomings of mitochondrial DNA analysis for cheetah conservation management"

René Meißner[1,2*], Sven Winter[1], Uta Westerhüs[3], Alexander Sliwa[4], Carola Greve[5], Lena Godsall Bottriell[6], Paul Bottriell[6,†], Carlos Rodríguez Fernandes[7,8], Paul Vercammen[9], Luke T. B. Hunter[10,11], Alexei V. Abramov[12], Leili Khalatbari[13,14,15,16] , Petr Horin[16,17], Pamela A. Burger[1], Stefan Prost[18,19,20,21*]

[1]Research Institute of Wildlife Ecology, University of Veterinary Medicine, Savoyenstraße 1, 1160 Vienna, Austria

[2]Institute for Ecology, Evolution and Diversity, Goethe University, Max-von-Laue-Straße 13, 60438 Frankfurt am Main, Germany

[3]Opel-Zoo, von Opel Hessische Zoostiftung, Königsteinerstrasse 35, 61476 Kronberg im Taunus, Germany

[4]Kölner Zoo AG, Riehler Straße 173, 50735 Cologne, Germany

[5]LOEWE Centre for Translational Biodiversity Genomics (LOEWE-TBG), Senckenberganlage 25, 60325 Frankfurt am Main, Germany

[6]Rex Foundation, White Rock House, 2 Whipsnade Road, Dunstable LU6 2NB, United Kingdom

[7]CE3C - Centre for Ecology, Evolution and Environmental Changes & CHANGE - Global Change and Sustainability Institute, Departamento de Biologia Animal, Faculdade de Ciências, Universidade de Lisboa, 1749-016 Lisboa, Portugal

[8]Faculdade de Psicologia, Universidade de Lisboa, Alameda da Universidade, 1649-013 Lisboa, Portugal

[9]Breeding Centre for Endangered Arabian Wildlife, Sharjah, United Arab Emirates

[10]Wildlife Conservation Society, New York, NY, USA

[11]School of Life Sciences, University of KwaZulu-Natal, Durban, South Africa

[12]Zoological Institute, Russian Academy of Sciences, Saint Petersburg, Russia

[13]CIBIO, Centro de Investigação Em Biodiversidade e Recursos Genéticos, InBIO Laboratório Associado, Campus de Vairão, Universidade Do Porto, 4485-661 Vairão, Portugal.

[14]BIOPOLIS Program in Genomics, Biodiversity and Land Planning, CIBIO, Campus de Vairão, 4485-661 Vairão, Portugal.

[15]Mohitban Society, No. 91, Moghaddas Ardebili str., Tehran 19859-14747, Iran.

[16]Department of Animal Genetics, University of Veterinary Sciences, Brno, Czech Republic

[17]Central European Institute of Technology, University of Veterinary Sciences Brno (CEITEC Vetuni), Brno, Czech Republic

[18]Konrad Lorenz Institute of Ethology, Vetmeduni, Savoyenstraße 1, 1160 Vienna, Austria

[19]Department of Behavioral and Cognitive Biology, University of Vienna, Djerassiplatz 1, 1030 Vienna, Austria

[20]Natural History Museum, Vienna, Central Research Laboratories, Burgring 7, 1010 Vienna, Austria

[21]South African National Biodiversity Institute, National Zoological Garden, 232 Boom Street, Pretoria 0002, South Africa

[†]We deeply regret the passing of our esteemed colleague Paul Bottriell during the course of the project.

*Corresponding authors: rene.meissner@vetmeduni.ac.at, stefanprost.research@protonmail.com

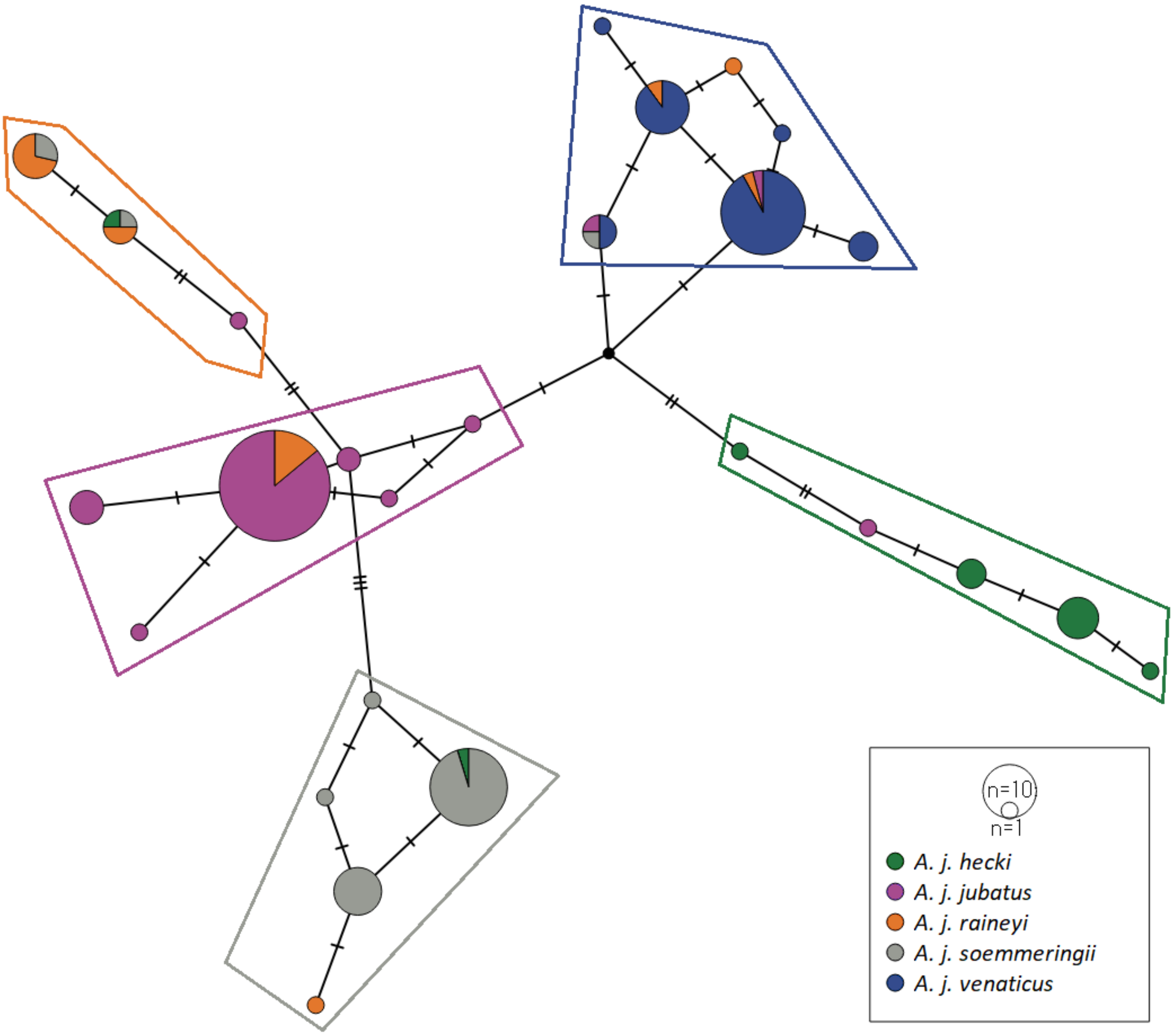

Supplementary figure 1: Median-joining network for 153 cheetah individuals based on the full sequences of the Cheetah Subspecies-specific Amplicon (CSA1-5); Pie-chart diameters represent sample sizes supporting a specific haplotype, Pie-chart colors represents subspecies assignment based on sample origin, encircles represent suggested subspecies haplogroup; mutations between haplotypes are indicated by dashes.

Supplementary table 1: Complete list of all 263 individual samples with detailed information (including origin, geographical origin-based subspecies assignment, age and conducted analysis).

Lab Code (A.j.)	Geographic origin-based subspecies assignment	Collection date	Country	Location	Voucher ID	Museum	CSA Illumina	ARMS
001	*A. j. raineyi*	1987	Kenya	Not on museum record	BMBC 300 612	Booth Mus. Natural History, UK		
003	*A. j. hecki*	1988	Burkina Faso	Humani Ranch nr. Birchenough Bridge – 19º57'S 32º20'E	BF. REX.1988	Nazinga Game Ranch, Pô	x	
004	*A. j. raineyi*	1987	Kenya	Not on museum record	BMBC 300 613	Booth Mus. Natural History, UK		
006	*A. j. jubatus*	1945	Zimbabwe	Manicaland south -19ºS 32ºE	ARCH. 2. 1945	Private		
007	*A. j. soemmeringii*	1830	Sudan	Kordofan region - 11ºN 30ºE	RMNH.MAM.51700	Naturalis, Netherlands		
008	*A. j. jubatus*	1910	South Africa	Not on museum record	RMNH.MAM.51702	Naturalis, Netherlands		
010	*A. j. raineyi*	1944	Kenya	Not on museum record	NMINH 1946.40.1	National Museum Ireland, Ireland		
011	*A. j. venaticus*	1893	Iran	Abadeh, south of Esfahan - 31º08'N 52º36'E	NMINH: 1893.139.1	National Museum Ireland, Ireland		
012	*A. j. soemmeringii*	1925	Chad	Luri, Mau-Masakory Rd., Kanem District -14º50'N 15º18'E	PCM. NN. 81	Powell-Cotton Museum Quex Park, UK	x	
013	*A. j. jubatus*	1936	Angola	Not on museum record	PCM. ANG. 1936	Powell-Cotton Museum Quex Park, UK		
017	*A. j. jubatus*	1921	Angola	Between Benguela/Bié/Moxico provs. - 12º25'S 14ºE	PCM. ANG. 95	Powell-Cotton Museum Quex Park, UK	x	
018	*A. j. raineyi*	1958	Kenya	Not on museum record	RMNH.MAM.16519	Naturalis, Netherlands		
019	*A. j. soemmeringii*	1930	Ethiopia	Not on museum record	MMS. 195a	Missie Museum Steyl, Netherlands		
020	*A. j. raineyi*	1936	Kenya	nr. Nairobi - 1°17'S 36°48'E	ZMA.MAM.9406	Naturalis, Netherlands		
021	*A. j. hecki*	1830	Senegal	Not on museum record	RMNH.MAM.51704	Naturalis, Netherlands	x	

023	*A. j. venaticus*	1840	India	Not on museum record	RMNH.MAM.51699	Naturalis, Netherlands		
024	*A. j. raineyi*	1972	Tanzania	Arusha District - 4ºS 37ºE	RMNH.MAM.41212	Naturalis, Netherlands		
025	*A. j. raineyi*	1900	Kenya	Not on museum record	NMW.Z.1912.014.4	National Museum Wales, UK	x	
026	*A. j. raineyi*	1939	Tanzania	Singida District, Manyoni – 5º45'S 34º50'E	RCSOM/A 118.7	The Royal College of Surgeons of England, UK	x	
028	*A. j. raineyi*	1930	Tanzania	Not on museum record	RCSOM/A 118.1	The Royal College of Surgeons of England, UK	x	
030	*A. j. raineyi*	1945	Kenya	Not on museum record	RCSOM/G 70.23	The Royal College of Surgeons of England, UK		
031	*A. j. venaticus*	1878	India	Not on museum record	UMZC. No.: K.5442	Uni. Mus. Zoology Cambridge, UK		
032	*A. j. soemmeringii*	1925	Somalia	Not on museum record	BMBC 102 784	Booth Mus. Natural History, UK	x	
036	*A. j. hecki*	1920	Togo	"Togo" inscribed in Indian ink on skull - 7ºN 1ºE	NMR 9990-00944	Natural History Museum Rotterdam, Netherlands	x	
037	*A. j. raineyi*	1955	Kenya	Not on museum record	PM. ZSL.1	Pat & Mary Morris collection, UK	x	
038	*A. j. venaticus*	1880	India	Not on museum record	WML.1963. 173. 104	World Museum Liverpool, UK		
039	*A. j. venaticus*	1880	India	Not on museum record	WML. 1963. 173. 105	World Museum Liverpool, UK	x	
042	*A. j. hecki*	1931	Algeria	Southern Oran	BMNH ZD 1939.1685	Natural History Museum London, UK	x	
043	*A. j. venaticus*	1992	Iran	Kavir Natl. Pk (Dasht-e Kavir) sth.of Tehran – 34º40'N 54º30'E	DKIU. 1.1992	Institut für Haustierkunde Universität Kiel, Germany		x
044	*A. j. soemmeringii*		Sudan/Somalia	Not on museum record	ID8339	CE3c; Breeding Centre for Endangered Arabian Wildlife, UAE	x	
045	*A. j. raineyi*		Sudan/Somalia	Not on museum record	ID8436	CE3c; Breeding Centre for Endangered Arabian Wildlife, UAE	x	
046	*A. j. soemmeringii*		Sudan/Somalia	Not on museum record	ID1873	CE3c; Breeding Centre for Endangered Arabian Wildlife, UAE	x	
047	*A. j. soemmeringii*		Sudan/Somalia	Not on museum record	ID8337	CE3c; Breeding Centre for Endangered Arabian Wildlife, UAE	x	

048	*A. j. soemmeringii*		Sudan/Somalia	Not on museum record	ID8338	CE3c; Breeding Centre for Endangered Arabian Wildlife, UAE	x	
050	*A. j. soemmeringii*		Sudan/Somalia	Not on museum record	ID8340	CE3c; Breeding Centre for Endangered Arabian Wildlife, UAE	x	
051	*A. j. soemmeringii*		Sudan/Somalia	Not on museum record	ID8343	CE3c; Breeding Centre for Endangered Arabian Wildlife, UAE	x	
053	*A. j. soemmeringii*		Sudan/Somalia	Not on museum record	ID6544	CE3c; Breeding Centre for Endangered Arabian Wildlife, UAE	x	
055	*A. j. soemmeringii*		Sudan/Somalia	Not on museum record	Kanu	CE3c; Breeding Centre for Endangered Arabian Wildlife, UAE	x	
057	*A. j. soemmeringii*		Sudan/Somalia	Not on museum record	Puppy	CE3c; Breeding Centre for Endangered Arabian Wildlife, UAE	x	
058	*A. j. soemmeringii*		Sudan/Somalia	Not on museum record	Shanu	CE3c; Breeding Centre for Endangered Arabian Wildlife, UAE	x	
060	*A. j. soemmeringii*		Sudan/Somalia	Not on museum record	Al Wabra 27	CE3c; Breeding Centre for Endangered Arabian Wildlife, UAE	x	
063	*A. j. venaticus*		Iran	Not on museum record	Iran.B.1	CE3c; Breeding Centre for Endangered Arabian Wildlife, UAE	x	
064	*A. j. venaticus*		Iran	Not on museum record	Iran.K.2	CE3c; Breeding Centre for Endangered Arabian Wildlife, UAE	x	
065	*A. j. venaticus*	1820	Iran	Chandannagar, Bengal - 22º51'N 88º 21'E	MNHN. A-7920	Mus. National d'Histoire Naturelle Paris, France		
066	*A. j. venaticus*	1828	India	Sirsa, Haryana State - 29°30'N 75°04'E	MNHN. A7919	Mus. National d'Histoire Naturelle Paris, France	x	
068	*A. j. soemmeringii*	1899	Ethiopia	High plateau country - 10ºN 38ºE	MNHN. 1901-541	Mus. National d'Histoire Naturelle Paris, France		
069	*A. j. raineyi*	1913	Kenya	Amala R. Valley - central Gregory Rift – 0º60'S 35º20'E	MNHN 1975-135	Mus. National d'Histoire Naturelle Paris, France		
070	*A. j. jubatus*	1920	Angola	Cubango Province - 16°S 19°E	MNHN 1921-142	Mus. National d'Histoire Naturelle Paris, France	x	
072	*A. j. soemmeringii*	1949	Chad	Fort Archambault (Sarh) - 9º07N 18º23E	MNHN 2006-460	Mus. National d'Histoire Naturelle Paris, France	x	

073	*A. j. jubatus*	1920	Angola	Not on museum record – probably Cubango Prov.	MNHN. 1962-2952	Mus. National d'Histoire Naturelle Paris, France		
074	*A. j. hecki*	1970	Western Sahara	80kms sth. Bir Anzarene – 23º54'N 14º32'W	CSIC.19316	CSIC Donana – Seville HQ, Spain		
075	*A. j. hecki*	1971	Western Sahara	80kms sth. Bir Anzarene – 23º54'N 14º32'W	CSIC.29888	CSIC Donana – Seville HQ, Spain	x	x
076	*A. j. hecki*	1972	Western Sahara	80kms sth. Bir Anzarene – 23º54'N 14º32'W	CSIC. 29889	CSIC Donana – Seville HQ, Spain	x	
077	*A. j. hecki*	1940	Western Sahara	Not on museum record	CSIC. 29890	CSIC Donana – Seville HQ, Spain		
078	*A. j. hecki*	1943	Algeria	El Gaada, Mascara Prov. - 35ºN 0ºE	CSIC. 29951	CSIC Donana – Seville HQ, Spain	x	
079	*A. j. venaticus*	1949	Afghanistan	Farah Province - 62ºN 32ºE	CN 4520	Zoological Museum Univ.Copenhagen, Denmark	x	
080	*A. j. raineyi*	1935	Kenya	South Masai Mara Reserve – 1.5ºS 35º5'E	CN 3493	Zoological Museum Univ.Copenhagen, Denmark	x	
081	*A. j. soemmeringii*	1947	Sudan	East. Equatoria, bet. Juba / Lowelli – 6ºN 33ºE	CN 5406	Zoological Museum Univ.Copenhagen, Denmark	x	
082	*A. j. jubatus*	1927	Zimbabwe	Manicaland South, Bikita dist. - 20º06'S 31º41'E	NMSA 864	KwaZulu Natal Mus. Pietermaritzburg, South Africa	x	
083	*A. j. jubatus*	1903	South Africa	Zululand, KwaZula Natal - 30ºS 28ºE	NMSA 865	KwaZulu Natal Mus. Pietermaritzburg, South Africa		
084	*A. j. jubatus*	1960	Botswana	Shashe R.-Tuli-Circle-Limpopo R. enclave – 22ºS 29ºE	RBIVY.REX.1	Private		
085	*A. j. jubatus*	1924	Namibia	Osohama, Etosha Pan – 18º32'S 16º49'E	KM 14429	Amathole Museum King Williams Town, South Africa	x	
086	*A. j. jubatus*	1931	Namibia	Maschi River, Caprivi Strip - 17º90'S 23º20'E	KM 14430	Amathole Museum King Williams Town, South Africa	x	
088	*A. j. jubatus*	1945	Namibia	Sth-central dist., Namaland nr. Asab – 25º22'S 17º55'E	KM 14432	Amathole Museum King Williams Town, South Africa	x	
089	*A. j. jubatus*	1925	Namibia	East-central dist., nr. Gobabis - 22º27'S – 18º58'E	KM 14433	Amathole Museum King Williams Town, South Africa		
090	*A. j. jubatus*	1970	Botswana	nr. Kang, Kgalagadi District – 23º70'S 22º50'E	BNM E4978	Botswana National Museum Gaborone. Botswana	x	
091	*A. j. jubatus*	1960	Botswana	Gweta / Rakops district – 21ºS 25ºE	CF.REX	Private		
093	*A. j. raineyi*	1920	Tanzania	Not on museum record	BMNH 1975.2147	Natural History Museum London, UK		

095	*A. j. jubatus*	1882	South Africa	Karoo, nr. Beaufort West – 32º20'S 22º36'E,	BMNH ZD 1890.2.26.1	Natural History Museum London, UK	x	
096	*A. j. jubatus*	1884	South Africa	Speared / shot Karoo, nr. Beaufort West – 32º20'S 22º36'E	BMNH. ZD 1886.1.25.1	Natural History Museum London, UK	x	
097	*A. j. jubatus*	1905	Malawi	Angoniland (adj. Planalto de Angonia Moz.) - 14ºS 34ºE	BMNH ZD 1905.8.13.1	Natural History Museum London, UK		
098	*A. j. jubatus*	1910	Democratic Republic Congo	Katanga Prov., nr. Luapula R - 9º30'S 28º33'E	BMNH ZD 1912.5.10.1	Natural History Museum London, UK	x	
099	*A. j. raineyi*	1947	Uganda	Buganda Province - 0º20'N 32º32'E	BMNH 1935.2.3.1	Natural History Museum London, UK	x	
102	*A. j. jubatus*	1928	Zambia	Not on museum record	BMNH 1935.9.1.134	Natural History Museum London, UK	x	
103	*A. j. jubatus*	1905	Angola	Not on museum record	BMNH ZD 1905.5.9.12	Natural History Museum London, UK	x	
104	*A. j. jubatus*	1931	Zambia	Broken Hill (now Kabwe) District – 14º27'S 28º27'E	BMNH ZD 1932.9.1.123	Natural History Museum London, UK	x	
105	*A. j. jubatus*	1815	South Africa	Cape of Good Hope area, Western Cape – 34ºS 19ºE	BMNH ZD 1981.742	Natural History Museum London, UK	x	
106	*A. j. jubatus*	1902	South Africa	Deelfontein (nth. of Karoo) – 30º59'S 23º48'E	BMNH ZD 1981.743	Natural History Museum London, UK		
107	*A. j. hecki*	1940	Nigeria	Northern Nigeria – 12ºN 9ºE	BMNH 1943.55	Natural History Museum London, UK		
108	*A. j. hecki*	1930	Nigeria	Yantumaki, Katsina Division – 12ºN 9ºE	BMNH 1932.12.27.1	Natural History Museum London, UK	x	x
109	*A. j. hecki*	1912	Burkina Faso	Manga See (Lake), east of Parc Natl de Pô - 11º40'N 1º06'W	ZMB MAM 56127	Museum für Naturkunde Berlin, Germany		
110	*A. j. hecki*	1898	Tunisia	Tunisian Sahara – 31ºN 10ºE	ZMB MAM 56276	Museum für Naturkunde Berlin, Germany		
111	*A. j. soemmeringii*	1821	Sudan	East-central – Aswan / Red Sea District – 21ºN 34ºE	ZMB MAM 1219	Museum für Naturkunde Berlin, Germany		
116	*A. j. hecki*	1904	Libya	Tripoli (Tarabulus) area – 32ºN 13ºE	ZMB MAM 56121	Museum für Naturkunde Berlin, Germany		
117	*A. j. hecki*	1904	Libya	Tripoli (Tarabulus) " "as above " "	ZMB MAM 13076	Museum für Naturkunde Berlin, Germany		
118	*A. j. soemmeringii*	1860	Sudan	Sennar State - bet. Blue / White Niles – 13ºN 33ºE	ZMB MAM 72847	Museum für Naturkunde Berlin, Germany	x	

119	*A. j. raineyi*	1909	Tanzania	South Tabora admin. Region – 6ºS 33ºE	ZMB MAM 56305	Museum für Naturkunde Berlin, Germany	x	
120	*A. j. raineyi*	1900	Tanzania	Kilimanjaro district - 4ºS 37ºE	ZMB MAM 56297	Museum für Naturkunde Berlin, Germany	x	
122	*A. j. raineyi*	1918	Tanzania	nr. Lake Balangida, Hanang area – 5ºS 35ºE	ZMB MAM 28991	Museum für Naturkunde Berlin, Germany		
124	*A. j. hecki*	1898	Western Sahara	Rio de Oro at coll. (precise dist. not recorded) 24ºN 14ºW	ZMB MAM 42242	Museum für Naturkunde Berlin, Germany	x	
125	A. j. venaticus	1912	Jordan	Wadi Musa – nr. Petra – 30º19'N 35º30'E	ZMB MAM 56122	Museum für Naturkunde Berlin, Germany	x	
126	*A. j. jubatus*	1901	Namibia	nr. Gt.Spitzkoppe, Erongo District – 22ºS 15ºE	ZMB MAM 91304	Museum für Naturkunde Berlin, Germany	x	
127	*A. j. venaticus*	1939	Iran	Not on museum record	BMNH 3666	Natural History Museum London, UK		
129	*A. j. venaticus*	1946	Turkmenistan	Mt Kosha-Chunga area – 35º28'N 61º58'E	ZMMU S-47281	Zoological Museum MSU Moscow, Russia		
130	*A. j. venaticus*	1941	Turkmenistan	Gt. Balhan-Dalgary foothills– 38º50'N 54º54'E	ZMMU S-49247	Zoological Museum MSU Moscow, Russia	x	
131	*A. j. venaticus*	1946	Turkmenistan	Mt Kosha-Chunga area – 35º28'N 61º58'E	ZMMU S-51711	Zoological Museum MSU Moscow, Russia		x
132	*A. j. venaticus*	1948	Turkmenistan	Chakmaklychanga, SE of Badkyz Res. - 35º30'N 62º24'E	ZMMU S-51898	Zoological Museum MSU Moscow, Russia		
133	*A. j. venaticus*	1880	Kazakhstan	SouthTranscaspian (Caspian Depression) – 46ºN 54ºE	ZMMU S-94398	Zoological Museum MSU Moscow, Russia		
134	*A. j. venaticus*	1858	Russia	Orenburg / Ural'sk Prov. - 49ºN 58ºE	ZIN 1891	Zool. Institute Russian Acad. Sciences St. Petersburg, Russia	x	
135	*A. j. venaticus*	1884	Turkmenistan	Maryyskaya (Zakaspyisk) Prov. - 37º40'N 62ºE	ZIN 2808	Zool. Institute Russian Acad. Sciences St. Petersburg, Russia	x	
138	*A. j. venaticus*	1857	Russia	Orenburg locality - foothills of Urals – 52ºN 56ºE	ZIN 1133	Zool. Institute Russian Acad. Sciences St. Petersburg, Russia	x	
139	*A. j. venaticus*	1934	Turkmenistan	Chardzhou, Lebapskaya Prov. - 39º05'N 63º34'E	ZIN 23893	Zool. Institute Russian Acad. Sciences St. Petersburg, Russia	x	
141	*A. j. hecki*	1886	Syria	Aleppo area – border reg. with Turkey – 36ºN 37ºE	CCEC40000366	Musée des Confluences Lyon, France		
143	*A. j. jubatus*	1970	Zimbabwe	Matebeleland South, West Nicholson – 21º04'S 29º22'E	NMMZ 56241	Natural History Museum Bulawayo, Zimbabwe	x	
144	*A. j. jubatus*		Zimbabwe	Mashonaland East - nr. Macheke – 18º05'S 31º50'E	NMMZ 59257	Natural History Museum Bulawayo, Zimbabwe	x	

145	*A. j. jubatus*	1960	Zimbabwe	Matabeleland, Bulawayo district – 20º06'S 28º35'E	NMMZ 17633	Natural History Museum Bulawayo, Zimbabwe	x	
147	*A. j. jubatus*	1967	Botswana	nr. Seruli, East Central District – 21º56'S 27º18'E	NMMZ 63600	Natural History Museum Bulawayo, Zimbabwe	x	
149	*A. j. jubatus*	1973	Zimbabwe	Mineral King Farm, nr. Matopos – 20º24'S 28º28'E	NMMZ 56283	Natural History Museum Bulawayo, Zimbabwe	x	x
151	*A. j. jubatus*	1970	Botswana	Kwando R./ Linyanti Swamp enclave - 18ºS 23ºE	NMMZ 55703	Natural History Museum Bulawayo, Zimbabwe	x	x
152	*A. j. jubatus*	1968	Botswana	East Central Dist., 16mls. west of Seruli – 22º19'S 27º29'E	NMMZ 63599	Natural History Museum Bulawayo, Zimbabwe	x	x
153	*A. j. jubatus*	1968	Botswana	Maun, Okavango Delta - 19º57'S 23º25'E	NMMZ 55706	Natural History Museum Bulawayo, Zimbabwe	x	x
154	*A. j. jubatus*	1969	Botswana	Kwaai R. Camp, Okavango East – 19º10'S 23º80'E	NMMZ 56281	Natural History Museum Bulawayo, Zimbabwe	x	
156	*A. j. jubatus*	1968	Botswana	Ngamiland, Gomoti R., Okavango east – 19º50'S 23º50'E	NMMZ 62826	Natural History Museum Bulawayo, Zimbabwe	x	
158	*A. j. jubatus*	1958	Zambia	Mazabuka vicinity – 15º52'S 27º45'E	NMMZ 8832	Natural History Museum Bulawayo, Zimbabwe	x	
159	*A. j. jubatus*	1970	Zimbabwe	Chipinge area, Manicaland – 20º09'S 32º37'E	NMMZ 59398	Natural History Museum Bulawayo, Zimbabwe	x	
160	*A. j. jubatus*	1975	Zimbabwe	Que Que (Kwekwe) Midlands – 18º56'S 29º48'E	NMMZ 55705	Natural History Museum Bulawayo, Zimbabwe	x	
161	*A. j. jubatus*	1968	Botswana	Okavango Delta region – 19º25'S 23ºE	NMMZ 62827	Natural History Museum Bulawayo, Zimbabwe	x	
163	*A. j. jubatus*	1966	Zimbabwe	Hartley (Chegutu), Mashonaland West – 18º09'S 30º07'E	NMMZ 59399	Natural History Museum Bulawayo, Zimbabwe	x	
164	*A. j. jubatus*	1972	Zimbabwe	West Nicholson, Matabeleland South – 21º 29ºE	NMMZ 56284	Natural History Museum Bulawayo, Zimbabwe	x	
166	*A. j. jubatus*	1970	Zimbabwe	nr. Kazuma Pan/Vic Falls Pk., Matabeleland – 18ºS 25ºE	NMMZ 53017	Natural History Museum Bulawayo, Zimbabwe	x	
167	*A. j. jubatus*	1974	Zimbabwe	Matabeleland Sth., West Nicholson – 21º03'S 29º20'E	NMMZ 57344	Natural History Museum Bulawayo, Zimbabwe	x	
169	*A. j. jubatus*		Zimbabwe	Not on museum record	NMMZ 58686	Natural History Museum Bulawayo, Zimbabwe	x	
170	*A. j. jubatus*		Zimbabwe	Ngezi District, nr. Sebakwe Dam, Midlands – 18º 30ºE	NMMZ 64768	Natural History Museum Bulawayo, Zimbabwe	x	

171	*A. j. jubatus*		Zimbabwe	Not on museum record	NMMZ 58698	Natural History Museum Bulawayo, Zimbabwe		
172	*A. j. jubatus*		Zimbabwe	Mashonaland East, Harare District – 17º49'S 31º04'E	NMMZ 59314	Natural History Museum Bulawayo, Zimbabwe	x	
176	*A. j. hecki*	1900	Syria	Golan Heights / Dar'a Prov towards Palestine	MCZ 16714	Museum Comparative Zoology Harvard, USA	x	
189	*A. j. raineyi*	1939	Ethiopia	Gondaraba / Che'w Bahir (nr. Stephanie Res) - 4º58'N 36º49'E	MSNG 35224	Civic Natural History Museum "Giacomo Doria" Genoa, Italy	x	
190	*A. j. raineyi*	1937	Ethiopia	Mega Escarpment, Boran Prov - 4º0'N 38º20'E	MSNG 33059a	Civic Natural History Museum "Giacomo Doria" Genoa, Italy	x	x
191	*A. j. raineyi*	1937	Ethiopia	Mega Escarpment, Boran Prov. - 4º0'N 38º20'E	MSNG 33059b	Civic Natural History Museum "Giacomo Doria" Genoa, Italy	x	
193	*A. j. hecki*	1855	Algeria	Not on museum record	MZUF 532	Museo "La Specola" Uni. di Florence, Italy		
194	*A. j. raineyi*	1952	Somalia	far south, Jilib (Gelib) area, Jubbada Dhexe Prov. - 0º29'N 42º46'E	MZUF 1664	Museo "La Specola" Uni. di Florence, Italy		x
204		1961		Not on museum record	MCZR 243	Museo Civico di Zoologia Rome, Italiy		
211		1925		Not on museum record	NMB 3562	Naturhistorisches Museum Basel, Switzerland		
212		1938		recorded as 'South Asian' origin	NMB 5234 / NMB 9180	Naturhistorisches Museum Basel, Switzerland		
221		1924		Not on museum record	MHNG 832.078	Muséum d'histoire naturelle Geneva, Switzerland		
222		1864		Not on museum record	MHNG 604.089	Muséum d'histoire naturelle Geneva, Switzerland		
223		1876		Not on museum record	UN 375	Finnish Museum of Natural History LUOMUS Helsinki Uni., Finland		
224		1980		Not on museum record	UN 1102	Finnish Museum of Natural History LUOMUS Helsinki Uni., Finland		
225		1967		Not on museum record	UN 1755	Finnish Museum of Natural History LUOMUS Helsinki Uni., Finland		
226				Not on museum record	UN 2295	Finnish Museum of Natural History LUOMUS Helsinki Uni., Finland		
227		1876		Not on museum record	UN 2303	Finnish Museum of Natural History LUOMUS Helsinki Uni., Finland		

228	*A. j. raineyi*	1911	Kenya	nr. Nairobi - 1°17'S 36°49'E	UN 2304	Finnish Museum of Natural History LUOMUS Helsinki Uni., Finland		
229				Not on museum record	UN 2305	Finnish Museum of Natural History LUOMUS Helsinki Uni., Finland		
237	*A. j. jubatus*	1992	Namibia	Not on museum record	NMBE 1023746	Natural History Museum der Bürgergemeinde Bern, Switzerland	x	
239	*A. j. raineyi*	1898	Somalia	Berbera/Guban Plain (off Gulf of Aden)" at 10ºN 45ºE	M1195	South Australian Museum Adelaide, Australia	x	
241		1925		Not on museum record	AMNH 70549	American Museum of Natural History New York, USA		
243	*A. j. jubatus*	1925	Angola	Bié Prov. bet. N'Harea & Reserva do Luando - 11ºS 17ºE	AMNH 80619	American Museum of Natural History New York, USA	x	
244	*A. j. jubatus*	1925	Angola	Bié Prov. bet. N'Harea & Reserva do Luando - 11ºS 17ºE	AMNH 80618	American Museum of Natural History New York, USA		
245	*A. j. jubatus*	1925	Angola	Huambo Province – 13ºS 16ºE	AMNH 80865	American Museum of Natural History New York, USA		
246	*A. j. jubatus*	1940	Mosambique	Inhaminga area, Sofala Province - 18º50'S 35º50'E	AMNH 119657	American Museum of Natural History New York, USA		
247	*A. j. jubatus*	1940	Mosambique	Inhaminga area, Sofala Province - 18º50'S 35º50'E	AMNH 119656	American Museum of Natural History New York, USA		
248	*A. j. jubatus*	1950	Botswana	nr. Dukwe/Bakwena Res., Francistown Dist. - 20º30'S 26º50'E	AMNH 169109	American Museum of Natural History New York, USA	x	
249	*A. j. jubatus*	1950	Botswana	nr. Dukwe/Bakwena Res., Francistown Dist. - 20º30'S 26º50'E	AMNH 169108	American Museum of Natural History New York, USA	x	
250	*A. j. raineyi*	1924	Tanzania	Ikoma area (100+kms east Lake Victoria) - 2º04'S 34º38E	AMNH 54352	American Museum of Natural History New York, USA	x	
251	*A. j. raineyi*	1946	Tanzania	Arusha Natl. Pk area, 40kms east Ngorongoro Cr. - 3º25'S 36ºE	AMNH 161139	American Museum of Natural History New York, USA		
252	*A. j. soemmeringii*	1929	Ethiopia	Arsi Prov, Awash R. region, Gt. Rift - 8º59'N 40º10'E	AMNH 81047	American Museum of Natural History New York, USA	x	
253	*A. j. venaticus*	1933	India	state / region / district unrecorded	AMNH 100309	American Museum of Natural History New York, USA	x	
254	*A. j. jubatus*	1940	Mosambique	Tambara vicinity, nth. Manicaland - 16º45'S 34º15'E	AMNH 119655	American Museum of Natural History New York, USA		

255	*A. j. jubatus*	1925	Angola	Bié Prov. bet. N'Harea & Reserva do Luando - 11ºS 17ºE	AMNH 80866	American Museum of Natural History New York, USA	x	
256	*A. j. raineyi*	1938	Kenya	bankside Sand R. nr. Keerkerok airstrip - 1º60'S 35º20'E	AMNH 114517	American Museum of Natural History New York, USA		
258	*A. j. raineyi*	1935	Tanzania	Arusha region - 4ºS 36ºE	AMNH 130135	American Museum of Natural History New York, USA		
259	*A. j. jubatus*	1940	Mosambique	Tambara vicinity, Nth Manicaland - 16º45'S 34º15'E	AMNH 119654	American Museum of Natural History New York, USA		
260	*A. j. soemmeringii*	1903	Somalia	Not on museum record	MZS Mam 03123	Musees De La Ville de Strasbourg, France		
261	*A. j. soemmeringii*	1903	Somalia	Not on museum record	MZS Mam 03124	Musees De La Ville de Strasbourg, France		
268	*A. j. raineyi*	1931	Kenya	Rift Valley Province, Masai Res. - 1º30'S 35ºE	YPM 9566	Peabody Museum of Natural History Yale Uni., USA		
269		1954		Not on museum record	YPM 13547	Peabody Museum of Natural History Yale Uni., USA		
270		1964		Not on museum record	UN 2302	Finnish Museum of Natural History LUOMUS Helsinki Uni., Finland		
271	*A. j. hecki*	1970	Nigeria	Yankari Game Reserve, Bauchi State - 9º45'N 10º30'E	YM CH 1	Yankari Game Reserve Museum, Nigeria		
278	*A. j. jubatus*		South Africa/Namibia	Not on museum record	Jeanette/#1463	Zoo La Palmyre, France	x	x
279	*A. j. jubatus*		South Africa/Namibia	Not on museum record	Hilda/#1921	Zoo Salzburg, Austria	x	
280	*A. j. soemmeringii*	1998	Somalia	Sharjah Wildlife Centre	Mahdi/ CF31 #6031	CE3c; Breeding Centre for Endangered Arabian Wildlife, UAE		
281	*A. j. soemmeringii*	1998	Somalia	Sharjah Wildlife Centre	Ziggy/ CF21 #4201	CE3c; Breeding Centre for Endangered Arabian Wildlife, UAE	x	x
286	*A. j. soemmeringii*	1999	Somalia	Sharjah Wildlife Centre	Scally/ CF19 #4206	CE3c; Breeding Centre for Endangered Arabian Wildlife, UAE		
287		2000		Sharjah Wildlife Centre	00 01C8 2420	CE3c; Breeding Centre for Endangered Arabian Wildlife, UAE		x

288	*A. j. soemmeringii*	1999	Somalia	Sharjah Wildlife Centre	Aisha/ CF17 #4229	CE3c; Breeding Centre for Endangered Arabian Wildlife, UAE	x	x
289	*A. j. soemmeringii*	1999	Somalia	Sharjah Wildlife Centre	Niswa/ CF16 #4216	CE3c; Breeding Centre for Endangered Arabian Wildlife, UAE	x	x
290	*A. j. soemmeringii*	1998	Somalia	Sharjah Wildlife Centre	00 01C7 2276	CE3c; Breeding Centre for Endangered Arabian Wildlife, UAE		x
292	*A. j. soemmeringii*	1999	Somalia	Sharjah Wildlife Centre	Shakes/ CF14 #4205	CE3c; Breeding Centre for Endangered Arabian Wildlife, UAE	x	x
293	*A. j. soemmeringii*	1999	Somalia	Sharjah Wildlife Centre	Annie/ CF13 #4215	CE3c; Breeding Centre for Endangered Arabian Wildlife, UAE	x	x
294	*A. j. soemmeringii*	1998	Somalia	Sharjah Wildlife Centre	Olive/ CF12 #4223	CE3c; Breeding Centre for Endangered Arabian Wildlife, UAE		x
295	*A. j. soemmeringii*	1998	Somalia	Sharjah Wildlife Centre	Oliver (01F127 ED)/CF11 #4202	CE3c; Breeding Centre for Endangered Arabian Wildlife, UAE	x	x
296	*A. j. soemmeringii*	1998	Somalia	Sharjah Wildlife Centre	Aziz/ CF10 #4208	CE3c; Breeding Centre for Endangered Arabian Wildlife, UAE		x
300	*A. j. soemmeringii*		Somalia	Sharjah Wildlife Centre	Marley/ CF09 #4203	CE3c; Breeding Centre for Endangered Arabian Wildlife, UAE		
301	*A. j. soemmeringii*		Somalia	Sharjah Wildlife Centre	Li (02C7 17B8)/ CF24 #4207	CE3c; Breeding Centre for Endangered Arabian Wildlife, UAE		
302	*A. j. soemmeringii*	2001	Somalia	Sharjah Wildlife Centre	Skebenga/ CF08 #4501	CE3c; Breeding Centre for Endangered Arabian Wildlife, UAE	x	x
303	*A. j. soemmeringii*		Somalia	Sharjah Wildlife Centre	Tokalosh/ CF07 #4500	CE3c; Breeding Centre for Endangered Arabian Wildlife, UAE		x
304	*A. j. soemmeringii*		Somalia	Sharjah Wildlife Centre	Sangoma/ CF06 #4499	CE3c; Breeding Centre for Endangered Arabian Wildlife, UAE		
305	*A. j. soemmeringii*	2001	Somalia	Sharjah Wildlife Centre	Somali/ CF05 #4472	CE3c; Breeding Centre for Endangered Arabian Wildlife, UAE	x	x
306	*A. j. soemmeringii*	2001	Somalia	Sharjah Wildlife Centre	Goliath/ CF04 #4473	CE3c; Breeding Centre for Endangered Arabian Wildlife, UAE	x	x
307	*A. j. jubatus*		Namibia	Sharjah Wildlife Centre	Gums/ CF03 #4268	CE3c; Breeding Centre for Endangered Arabian Wildlife, UAE	x	

308		1999		Sharjah Wildlife Centre	00-01C7-0706/ CF02 #4415	CE3c; Breeding Centre for Endangered Arabian Wildlife, UAE		x
309	*A. j. soemmeringii*	1999	Somalia	Sharjah Wildlife Centre	00-01C7-F346/ CF01 #4421	CE3c; Breeding Centre for Endangered Arabian Wildlife, UAE	x	
313	*A. j. soemmeringii*	1999	Somalia	Sharjah Wildlife Centre	Mitsy/ CF22 #4418	CE3c; Breeding Centre for Endangered Arabian Wildlife, UAE		
334	*A. j. jubatus*		South Africa	Not on museum record	NMW ST 554	Naturhistorisches Museum Wien, Austria		
337	*A. j. raineyi*		Tanzania	Not on museum record	ZMB 56289	Museum für Naturkunde Berlin, Germany		
338	*A. j. venaticus*		Iran	Not on museum record	AJIran 01	University of Veterinary Medicine Vienna, Austria		
339	*A. j. venaticus*		Iran	Not on museum record	AJIran 02	University of Veterinary Medicine Vienna, Austria	x	
340	*A. j. venaticus*		Iran	Not on museum record	AJIran 03	University of Veterinary Medicine Vienna, Austria		
341	*A. j. venaticus*		Iran	Not on museum record	AJIran 04	National Museum of Iran, Iran		
342	*A. j. venaticus*		Iran	Not on museum record	AJIran 05	National Museum of Iran, Iran		x
343	*A. j. venaticus*		Iran	Not on museum record	AJIran 06	National Museum of Iran, Iran		
344	*A. j. venaticus*		Iran	Not on museum record	AJIran 07	National Museum of Iran, Iran		
345	*A. j. venaticus*		Iran	Not on museum record	AJIran 08	National Museum of Iran, Iran		
347	*A. j. venaticus*		Iran	Not on museum record	AJIran 10	National Museum of Iran, Iran	x	
349	*A. j. venaticus*		Iran	Not on museum record	AJIran M1A	University of Veterinary Medicine Vienna, Austria	x	x
350	*A. j. venaticus*		Iran	Not on museum record	AJIran M2A	University of Veterinary Medicine Vienna, Austria	x	x
351	*A. j. raineyi*		Kenya	Not on museum record	Claudia	Private	x	
353	*A. j. hecki*		Western Sahara	Not on museum record	MTD B13054	Museum für Tierkunde Dresden, Deutschland		
354	*A. j. hecki*		Libya	Not on museum record	NMW 12070	Naturhistorisches Museum Wien, Austria	x	x

355	*A. j. hecki*		Libya	Not on museum record	NMW 12071	Naturhistorisches Museum Wien, Austria		
356	*A. j. hecki*		Libya	Not on museum record	NMW 12072	Naturhistorisches Museum Wien, Austria	x	x
357	*A. j. hecki*		Mali	Not on museum record	NMW 2880	Naturhistorisches Museum Wien, Austria		
358	*A. j. jubatus*		Democratic Republic Congo	04.43 s - 24.25 e	RMCA 10300	Royal Museum of Central Africa, Belgium		
359	*A. j. jubatus*		Democratic Republic Congo	07.04 s - 29.45 e / adult collected with RMCA12009	RMCA 12008	Royal Museum of Central Africa, Belgium		
360	*A. j. jubatus*		Democratic Republic Congo	07.04 s - 29.45 e / juvenile collected with RMCA12008	RMCA 12009	Royal Museum of Central Africa, Belgium		
361	*A. j. jubatus*		Democratic Republic Congo	12.50 s - 29.19 e	RMCA 1236	Royal Museum of Central Africa, Belgium		
362	*A. j. jubatus*		Democratic Republic Congo	06.30 s - 16.51 e / collected at the same date as RMCA13103	RMCA 13102	Royal Museum of Central Africa, Belgium		
363	*A. j. jubatus*		Democratic Republic Congo	06.30 s - 16.51 e / collected at the same date as RMCA13102	RMCA 13103	Royal Museum of Central Africa, Belgium		
364	*A. j. soemmeringii*		Central African Republic	04.21 n - 18.37 e	RMCA 17201	Royal Museum of Central Africa, Belgium		
365	*A. j. jubatus*		Democratic Republic Congo	10.49 s - 26.07 e	RMCA 18307	Royal Museum of Central Africa, Belgium		
367	*A. j. jubatus*		Democratic Republic Congo	12.45 s - 28.34 e	RMCA 19237	Royal Museum of Central Africa, Belgium		
368	*A. j. jubatus*		Democratic Republic Congo	08.44 s - 25.00 e	RMCA 22347	Royal Museum of Central Africa, Belgium	x	
369	*A. j. jubatus*		Democratic Republic Congo	06.28 s - 18.10 e	RMCA 22390	Royal Museum of Central Africa, Belgium		

370	*A. j. jubatus*		Democratic Republic Congo	10.12 s - 27.25 e	RMCA 454	Royal Museum of Central Africa, Belgium	x	
371	*A. j. jubatus*		Democratic Republic Congo	10.32 s - 27.54 e	RMCA 4998	Royal Museum of Central Africa, Belgium	x	
372	*A. j. jubatus*		Angola	12.34 s - 13.24 e	RMCA 6058	Royal Museum of Central Africa, Belgium		
375	*A. j. soemmeringii*		Sudan	Not on museum record	SMF 15756	Senckenberg Museum, Germany		
376	*A. j. hecki*		Cameroon	= 100km North of Maroua, North Cameroon	SMF 4632	Senckenberg Museum, Germany		
377	*A. j. hecki*		Egypt	Lat: 30.35 Long: 29.28333 (45km NE of EL Maghra)	SMF 58993	Senckenberg Museum, Germany	x	x
379	*A. j. hecki*		Egypt	Not on museum record	SMNS 18941	Staatliches Museum für Naturkunde Stuttgart, Germany		
380	*A. j. soemmeringii*		Djibouti	Not on museum record	SMNS 38432	Staatliches Museum für Naturkunde Stuttgart, Germany		
383	*A. j. soemmeringii*		Sudan	Not on museum record	SMNS 600	Staatliches Museum für Naturkunde Stuttgart, Germany		
384	*A. j. raineyi*		Kenya	Not on museum record	Tigger	Breeder	x	x
388	*A. j. raineyi*		Tanzania	Not on museum record	ZFMK 34.64	Zoologisches Forschungsmuseum Alexander Koenig, Germany		
390	*A. j. hecki*		Nigeria	Not on museum record	ZFMK 83.351	Zoologisches Forschungsmuseum Alexander Koenig, Germany		
391	*A. j. hecki*		Nigeria	Not on museum record	ZFMK 87.652	Zoologisches Forschungsmuseum Alexander Koenig, Germany		
394	*A. j. soemmeringii*	1930	Ethiopia	Not on museum record	ADJ 1	DECAN		
395	*A. j. soemmeringii*	1930	Ethiopia	Not on museum record	ADJ 2	DECAN		
396	*A. j. soemmeringii*	1972	Ethiopia	Not on museum record	ADJ 3	DECAN	x	
397	*A. j. raineyi*	1972	Ethiopia	Not on museum record	ADJ 4	DECAN		

399	*A. j. soemmeringii*	1930	Ethiopia	Not on museum record	ADJ 6	DECAN	x	x
400	*A. j. soemmeringii*	1930	Ethiopia	Not on museum record	ADJ 7	DECAN		
401	*A. j. soemmeringii*		Djibouti	Not on museum record	ADJ 8	DECAN		
402	*A. j. raineyi*		Tanzania	Not on museum record	ZMB 34306	Museum für Naturkunde Berlin, Germany		
403	*A. j. raineyi*		Tanzania	Not on museum record	ZMB 56306	Museum für Naturkunde Berlin, Germany		
404	*A. j. raineyi*		Tanzania	Not on museum record	ZMB 56302	Museum für Naturkunde Berlin, Germany		x
406	*A. j. raineyi*		Tanzania	Not on museum record	ZMB 56309	Museum für Naturkunde Berlin, Germany		
408	*A. j. raineyi*		Tanzania	Not on museum record	ZMB56128	Museum für Naturkunde Berlin, Germany		
484	*A. j. venaticus*	2015	Iran	Touran NP	AJV_008	University of Veterinary Medicine Vienna, Austria	x	
485	*A. j. venaticus*	2015	Iran	Dareh Anjir WR	AJV_017	University of Veterinary Medicine Vienna, Austria	x	
487	*A. j. venaticus*	2015	Iran	Naybandan WR	AJV_172	University of Veterinary Medicine Vienna, Austria	x	
489	*A. j. venaticus*	2015	Iran	Touran NP	AJV_223	University of Veterinary Medicine Vienna, Austria	x	
491	*A. j. venaticus*	2015	Iran	Dareh Anjir WR	AJV_276	University of Veterinary Medicine Vienna, Austria	x	x
492	*A. j. venaticus*	2015	Iran	Dareh Anjir WR	AJV_283	University of Veterinary Medicine Vienna, Austria	x	
494	*A. j. venaticus*	2015	Iran	Dareh Anjir WR	AJV_285	University of Veterinary Medicine Vienna, Austria	x	
496	*A. j. venaticus*	2015	Iran	Dareh Anjir WR	AJV_288	University of Veterinary Medicine Vienna, Austria	x	
498	*A. j. venaticus*	2015	Iran	Touran NP	AJV_308	University of Veterinary Medicine Vienna, Austria	x	
499	*A. j. venaticus*	2015	Iran	Not on museum record	AJV_312	University of Veterinary Medicine Vienna, Austria	x	

500	*A. j. venaticus*	2015	Iran	Touran NP	AJV_335	University of Veterinary Medicine Vienna, Austria	x	x
502	*A. j. venaticus*	2015	Iran	Touran NP	AJV_339	University of Veterinary Medicine Vienna, Austria	x	
503	*A. j. venaticus*	2015	Iran	Not on museum record	AJV_354	University of Veterinary Medicine Vienna, Austria	x	x
504	*A. j. venaticus*	2015	Iran	Miandasht WR	AJV_375	University of Veterinary Medicine Vienna, Austria	x	
505	*A. j. venaticus*	2015	Iran	Touran NP	AJV_KOOSHKI	University of Veterinary Medicine Vienna, Austria	x	x
506	*A. j. hecki*		Algeria	Not on museum record	DZ_1000	University of Veterinary Medicine Vienna, Austria	x	

Supplementary table 2: illumina adapter list for the CSA approach, including index ID, adapter type, adapter sequence, index sequence, illumina tail complement sequence and annealing temperature of the adapter PCR [TM[°C]].

ID	type	adapter seqeunce 5'-3'	index 5'-3'	illumina tail complement 5'-3'
F1	p5	AATGATACGGCGACCACCGAGATCTACAC	TAGATCGC	ACACTCTTTCCCTACACGA
F2	p5	AATGATACGGCGACCACCGAGATCTACAC	CTCTCTAT	ACACTCTTTCCCTACACGA
F3	p5	AATGATACGGCGACCACCGAGATCTACAC	TATCCTCT	ACACTCTTTCCCTACACGA
F4	p5	AATGATACGGCGACCACCGAGATCTACAC	AGAGTAGA	ACACTCTTTCCCTACACGA
F5	p5	AATGATACGGCGACCACCGAGATCTACAC	GTAAGGAG	ACACTCTTTCCCTACACGA
F6	p5	AATGATACGGCGACCACCGAGATCTACAC	ACTGCATA	ACACTCTTTCCCTACACGA
F7	p5	AATGATACGGCGACCACCGAGATCTACAC	AAGGAGTA	ACACTCTTTCCCTACACGA
F8	p5	AATGATACGGCGACCACCGAGATCTACAC	CTAAGCCT	ACACTCTTTCCCTACACGA
F9	p5	AATGATACGGCGACCACCGAGATCTACAC	TGAACCTT	ACACTCTTTCCCTACACGA
F10	p5	AATGATACGGCGACCACCGAGATCTACAC	TGCTAAGT	ACACTCTTTCCCTACACGA
F11	p5	AATGATACGGCGACCACCGAGATCTACAC	TAAGTTCC	ACACTCTTTCCCTACACGA

F12	p5	AATGATACGGCGACCACCGAGATCTACAC	ATAGAGGC	ACACTCTTTCCCTACACGA
F13	p5	AATGATACGGCGACCACCGAGATCTACAC	GGCTCTGA	ACACTCTTTCCCTACACGA
F14	p5	AATGATACGGCGACCACCGAGATCTACAC	AGGCGAAG	ACACTCTTTCCCTACACGA
F16	p5	AATGATACGGCGACCACCGAGATCTACAC	CAGGACGT	ACACTCTTTCCCTACACGA
R6	p7	CAAGCAGAAGACGGCATACGAGAT	TATACAAG	GTGACTGGAGTTCAGACGTG
R7	p7	CAAGCAGAAGACGGCATACGAGAT	ATGATCTG	GTGACTGGAGTTCAGACGTG
R8	p7	CAAGCAGAAGACGGCATACGAGAT	TACTCTAC	GTGACTGGAGTTCAGACGTG
R9	p7	CAAGCAGAAGACGGCATACGAGAT	ATAAGCTA	GTGACTGGAGTTCAGACGTG
R10	p7	CAAGCAGAAGACGGCATACGAGAT	TAGTATAG	GTGACTGGAGTTCAGACGTG
R11	p7	CAAGCAGAAGACGGCATACGAGAT	ATTACAAG	GTGACTGGAGTTCAGACGTG
R12	p7	CAAGCAGAAGACGGCATACGAGAT	TAATTGGC	GTGACTGGAGTTCAGACGTG
R13	p7	CAAGCAGAAGACGGCATACGAGAT	ATCTCTAC	GTGACTGGAGTTCAGACGTG
R14	p7	CAAGCAGAAGACGGCATACGAGAT	TAGATCTG	GTGACTGGAGTTCAGACGTG
R15	p7	CAAGCAGAAGACGGCATACGAGAT	ATGCGGAC	GTGACTGGAGTTCAGACGTG
R16	p7	CAAGCAGAAGACGGCATACGAGAT	TAATCAGT	GTGACTGGAGTTCAGACGTG
R17	p7	CAAGCAGAAGACGGCATACGAGAT	ATTTTCAC	GTGACTGGAGTTCAGACGTG
R18	p7	CAAGCAGAAGACGGCATACGAGAT	TACACTGT	GTGACTGGAGTTCAGACGTG
R19	p7	CAAGCAGAAGACGGCATACGAGAT	ATGGCCAC	GTGACTGGAGTTCAGACGTG
R20	p7	CAAGCAGAAGACGGCATACGAGAT	TACCGGTG	GTGACTGGAGTTCAGACGTG
R21	p7	CAAGCAGAAGACGGCATACGAGAT	ATCGAAAC	GTGACTGGAGTTCAGACGTG

Supplementary table 3: Presence-absence table of tested samples of the three possible bands occurring on agarose gel of the *A. j. soemmeringii* haplotype specific ARMS: 189 bp band (unspecific), 110 bp band (*A. j. soemmeringii* haplogroup specific), >1000 bp band (additional band observed in samples possessing the *A. j. soemmeringii* haplogroup).

Lab Code (AJ)	geographical origin-based subspecies assignment	189 bp band	110 bp band	>1000 bp band
043	*A. j. venaticus*	yes	no	no
108	*A. j. hecki*	yes	no	no
131	*A. j. venaticus*	yes	no	no
149	*A. j. jubatus*	yes	no	no
151	*A. j. jubatus*	yes	no	no
152	*A. j. jubatus*	yes	no	no
153	*A. j. jubatus*	yes	no	no
190	*A. j. soemmeringii*	yes	no	no
194	*A. j. jubatus*	yes	no	no
278	*A. j. jubatus*	yes	no	no
281	*A. j. soemmeringii*	yes	yes	yes
288	*A. j. soemmeringii*	yes	yes	yes
289	*A. j. soemmeringii*	yes	yes	yes
292	*A. j. soemmeringii*	yes	yes	yes
293	*A. j. soemmeringii*	yes	yes	yes
294	*A. j. soemmeringii*	yes	yes	yes
295	*A. j. soemmeringii*	yes	yes	yes
296	*A. j. soemmeringii*	yes	yes	yes
302	*A. j. soemmeringii*	yes	yes	yes
305	*A. j. soemmeringii*	yes	no	no
306	*A. j. soemmeringii*	yes	no	no

342	*A. j. venaticus*	yes	no	no
349	*A. j. venaticus*	yes	no	no
350	*A. j. venaticus*	yes	no	no
356	*A. j. hecki*	yes	yes	yes
377	*A. j. hecki*	yes	no	no
384	*A. j. raineyi*	yes	no	no
399	*A. j. soemmeringii*	yes	yes	yes
404	*A. j. jubatus*	yes	no	no
491	*A. j. venaticus*	yes	no	no
500	*A. j. venaticus*	yes	no	no
503	*A. j. venaticus*	yes	no	no
505	*A. j. venaticus*	yes	no	no

Supplementary table 4: CITES-registered institutions within this study and their registration numbers.

Institution	CITES Registration Number
Amathole Museum, King William's Town, South Africa	**ZA020**
Botswana National Museum, Gaborone, Botswana	**BW004**
cE3c – Centre for Ecology, Evolution and Environmental Changes, Faculdade de Ciências da Universidade de Lisboa, Portugal	**PT004**
Field Museum of Natural History, Chicago, USA	**US012**
Forschungsinstitut für Wildtierkunde und Ökologie, Vetmeduni Vienna, Österreich	**AT031**
Harvard University Museum of Comparative Zoology,Cambridge, 02138	**US051**
Koret School Veterinary Medicine, Hebrew University of Jerusalem, Israel	**IL002**
Kwa-Zulu Natal Museum, Pietermaritzburg, South Africa	**ZA025**
Leiden University, Institute of Biology Dept Integrative Zoology, Leiden, Netherlands	**NL001**
Museo Civico di Storia Naturale Giacomo Doria, Genova, Italy	**IT019**

Museum für Naturkunde Berlin, Berlin, Germany	**DE203-06**
Museum National d'Histoire Naturelle, Paris, France	**FR75A**
Museum of Evolution, Uppsala, Sweden	**SE010**
National Zoological Gardens of South Africa, Pretoria, South Africa	**ZA034**
Natural History Museum "La Specola", Florence, Italy	**IT008**
Natural History Museum Denmark, Copenhagen, Denmark	**DK003**
Natural History Museum of Geneva, Switzerland	**CH004**
Natural History Museum, London, England	**GB001**
Naturhistorisches Museum Basel, Switzerland	**CH002**
Naturmuseum Sankt Gallen, Switzerland	**CH033**
Real jardín botánico Consejo superior de investigaciones cientificas, Madrid, Spain	**ES001**
Tel Aviv University, Israel	**IL001**
Zoologisches Museum der Universität Zürich, Switzerland	**CH005**
